# A Poisson reduced-rank regression model for association mapping in sequencing data

**DOI:** 10.1186/s12859-022-05054-6

**Published:** 2022-12-08

**Authors:** Tiana Fitzgerald, Andrew Jones, Barbara E. Engelhardt

**Affiliations:** 1 Department of Computer Science, Princeton University, Princeton, NJ USA; 2 Data Science and Biotechnology Institute, Gladstone Institutes, San Francisco, CA USA; 3 Department of Biomedical Data Science, Stanford University, Stanford, CA USA

**Keywords:** Single-cell RNA-sequencing, Association mapping, Count-based models, Reduced-rank regression

## Abstract

**Background:**

Single-cell RNA-sequencing (scRNA-seq) technologies allow for the study of gene expression in individual cells. Often, it is of interest to understand how transcriptional activity is associated with cell-specific covariates, such as cell type, genotype, or measures of cell health. Traditional approaches for this type of association mapping assume independence between the outcome variables (or genes), and perform a separate regression for each. However, these methods are computationally costly and ignore the substantial correlation structure of gene expression. Furthermore, count-based scRNA-seq data pose challenges for traditional models based on Gaussian assumptions.

**Results:**

We aim to resolve these issues by developing a reduced-rank regression model that identifies low-dimensional linear associations between a large number of cell-specific covariates and high-dimensional gene expression readouts. Our probabilistic model uses a Poisson likelihood in order to account for the unique structure of scRNA-seq counts. We demonstrate the performance of our model using simulations, and we apply our model to a scRNA-seq dataset, a spatial gene expression dataset, and a bulk RNA-seq dataset to show its behavior in three distinct analyses.

**Conclusion:**

We show that our statistical modeling approach, which is based on reduced-rank regression, captures associations between gene expression and cell- and sample-specific covariates by leveraging low-dimensional representations of transcriptional states.

**Supplementary Information:**

The online version contains supplementary material available at 10.1186/s12859-022-05054-6.

## Background

Recent advances in high-throughput genomic assays have allowed for the creation of expansive data sets that are useful for exploring biological variation across cells. In particular, single-cell RNA-sequencing (scRNA-seq) technologies provide gene expression measurements at the individual cell level, allowing for the analysis of variation in transcriptional activity across cells within a single sample [1–4]. While characterizing this variation is useful by itself for exploratory analysis, it is also of interest to study in a more targeted way how variation relates to cell-specific covariates, such as cell type, genotype, and cell health. Studying associations between gene expression and properties of single cells has the potential to enrich our understanding of the relationship between these covariates and transcription at single-cell resolution.

While methods for association studies have been widely developed for bulk RNA-sequencing (RNA-seq) data [5, 6], methods for studying associations on the level of individual cells are much less developed. Moreover, there are several unique challenges in manipulating and analyzing the data generated by these single-cell assays, as compared to bulk RNA-seq assays. These scRNA-seq data sets are high dimensional—there are tens of thousands of genes in the human genome—making them difficult to interpret gene-by-gene; furthermore, the count-based nature of the data—made up of counts of sequenced RNA fragments that map to a specific gene in a genome to approximate expression levels of that gene—presents a challenge for many standard statistical tools with Gaussian assumptions [7].

In this paper, we propose a statistical modeling approach based on reduced-rank regression that captures associations between gene expression and cell- and sample-specific covariates by leveraging low-dimensional representations of transcription. Within this framework, we propose two specific models: Poisson reduced-rank regression (PRRR), which adapts a generalized linear model to the reduced rank setting, and nonnegative Poisson reduced-rank regression (nn-PRRR), which provides interpretable nonnegative regression components. In what follows, we first review several related threads of research and describe our modeling approach. Then, using simulated data and single-cell RNA-seq data, spatial gene expression data, and bulk RNA-seq data, we show that our models are useful for a wide range of association study types, including studying the transcriptional hallmarks of cell types, genes correlated with disease status, and expression QTLs.

### Genome-wide association studies

Since the completion of the Human Genome Project in 2003 [8] and the HapMap project in 2005 [9], researchers have developed the genomic and statistical tools necessary to study the human genome at a large scale in order to better detect, treat, and prevent diseases. Genome-wide association studies (GWAS) are used to identify disease-causing genetic variation across complete genomes. Genetic variation often comes in the form of single nucleotide polymorphisms (SNPs) that can be compared between healthy patients and patients with a disease [10]. GWAS have found a plethora of genetic variants that are associated with common diseases such as asthma, type 2 diabetes, and more [11, 12].

In a similar vein, quantitative trait loci (QTL) studies identify associations between genetic variants and quantitative phenotypes [13, 14]. A common experimental setup is to use gene expression levels as the quantitative phenotype, in which case the association is referred to as an expression QTL (eQTL) [15, 16]. Most eQTL studies have relied on bulk RNA-seq technologies to measure the gene expression levels of samples from tissues with heterogeneous cell types [17–19].

In this context, the statistical eQTL problem is to estimate the pairwise association between a set of genetic variants (the covariates or explanatory variables) and the expression level of each gene (the outcome variables). This is typically performed using a linear regression model. In particular, let ${\mathbf {X}}$ be an $N\times P$ matrix containing information about genetic variants across *P* SNPs for *N* individuals or tissue samples, and let ${\mathbf {Y}} \in {\mathbb {R}}^{N \times Q}$ be a matrix of corresponding gene expression levels across *Q* genes in these individuals or tissues  [7]. Generically, these approaches use a model of the form

1 $$\begin{aligned} {\mathbf {y}}_{\cdot q} = {\mathbf {x}}_{\cdot p} b_{pq} + \varvec{\epsilon }, \end{aligned}$$

where ${\mathbf {x}}_{\cdot p}$ is the *p*th column of ${\mathbf {X}}$, ${\mathbf {y}}_{\cdot q}$ is the *q*th column of ${\mathbf {Y}}$, $\varvec{\epsilon } \in {\mathbb {R}}^{N}$ is a vector of independent zero-mean Gaussian-distributed noise terms, and $b_{pq} \in {\mathbb {R}}$ is a scalar coefficient representing the linear relationship between SNP *p* and gene *q* for $p = 1, \dots , P$ and $q = 1,\dots , Q$. Downstream tests for significance can be performed on these coefficients to identify associations [20–22]. Without further assumptions, this model estimates the marginal association between single SNPs and single genes independently. To accommodate polygenic contributions to phenotypes, multivariate models of the form

2 $$\begin{aligned} {\mathbf {Y}} = {\mathbf {X}} {\mathbf {B}} + \varvec{\epsilon }, \end{aligned}$$

have been considered, where ${\mathbf {B}} \in {\mathbb {R}}^{P \times Q}$ is a matrix of coefficients [23–25]. Under this framework, sparsity-inducing priors for ${\mathbf {B}}$ have been proposed in order to scale these models to high-dimensional genotype data [26, 27].

The advent of scRNA-seq technologies has opened the door for narrowing the investigation of genotype-phenotype relationships from the level of whole tissues to the level of individual cells. However, existing computational tools are insufficient for this purpose: they typically do not accommodate count-based data, and they are seldom robust to high-dimensional outcome variables. It is difficult to control the hypothesis testing error rate in many eQTL analyses, which run millions to trillions of univariate association hypothesis tests (one for each SNP-gene pair) [7, 18, 19, 28, 29].

### Count-based models

A further drawback of existing association testing frameworks is their assumption of Gaussian-distributed data. Most canonical regression models assume an independent normally-distributed response variable, with $\varvec{\epsilon } \sim {\mathcal {N}}({\mathbf {0}}, \sigma ^2 {\mathbf {I}}_N)$ in Eq. (1). However, when the data consist of count-based measurements, as for RNA-seq data, this assumption may be problematic. Various transformations have been proposed to make the response variable approximately Gaussian [30, 31], but these transformations are known to distort the data distribution in undesirable ways [32–34]. Count-based scRNA-seq data is discrete and nonnegative, with many gene expression counts having a value of zero. The sparsity of the data poses an additional challenge to these standard transformations [32].

An alternative to this approach is to model the gene expression data with a discrete distribution. A common choice is the Poisson distribution, whose support is restricted to the nonnegative integers and has been shown to improve the representation and interpretation of scRNA-seq data when fitting statistical models [32, 35]. A recent approach using a Poisson data likelihood proposed a naive Bayes model that assigns cell-type identities to samples in scRNA-seq data based on reference data [36]. The model uses a Poisson distribution to represent the count-based data, but the high number of zeros in the data still poses a challenge. The sparsity of the data interferes with standard estimates such as maximum likelihood estimates as rates of zero can be produced for thousands of genes, making the model sensitive to genes with low expression counts [36]. The model handles this challenge by introducing a hierarchical structure, placing posterior distributions on parameters in order to recover non-zero rate estimates for genes with zero counts in the reference data. However, the naive Bayes model also assumes independence between genes, although this assumption does not hold in practice, as expression has been observed to be correlated between genes [32, 37, 38].

### Modeling multiple data modalities

Latent variable modeling approaches have also been proposed for modeling multi-view data. The most popular approach has been canonical correlation analysis (CCA, [39]) and its probabilistic variants [40–42]. CCA seeks a low-dimensional linear mapping of two paired data matrices such that the resulting low-dimensional projections of both matrices are maximally correlated.

$$\begin{aligned} \max \rho ({\mathbf {X}} {\mathbf {u}}, {\mathbf {Y}} {\mathbf {v}})~~~~\text {subject to } {\mathbf {u}}^\top {\mathbf {u}} = 1, {\mathbf {v}}^\top {\mathbf {v}} = 1, \end{aligned}$$

where $\rho$ is the Pearson correlation function. The probabilistic version of this model projects the features of each data modality into a shared low-dimensional latent space, assuming heteroskedastic residual errors, maximizing the amount of variance explained in the data modalities by the latent subspace. The weights, or factor loadings, in CCA models allow us to identify covarying features across data modalities. A formal connection between CCA and reduced-rank regression has been shown [43], where the canonical subspace found by CCA is the same as the subspace of the maximum likelihood estimator for the reduced-rank regression model. Despite their connections, the unsupervised nature of CCA does not lend itself directly to association mapping between the data modalities. Conversely, reduced-rank regression has a natural association testing framework because of its regression foundation.

Recently, a latent variable model based on latent Dirichlet allocation [44, 45] for jointly modeling gene expression and genotype was proposed [46]. This model projected both genotype data—using an equivalent of the Structure model [45]—and count-based gene expression data—using a telescoping LDA model [44]—onto a shared latent subspace; we may then identify covarying genes and genotypes in a nonnegative latent representation. But discovering associations in this framework requires association testing in held-out data, which is limited by existing univariate methods and population data.

### Reduced-rank regression approaches

The transcriptional states of cells tend to exhibit strong correlations between genes [47]. Thus, it is likely that the relationship between cell covariates and transcriptional phenotypes in scRNA-seq data need not be modeled gene-by-gene. Rather, it is reasonable to assume that these associations exhibit a low-dimensional structure. Furthermore, treating each gene as independent is computationally and statistically inefficient; we would like to exploit these relationships to perform fewer association tests and leverage shared variation to improve statistical power in these often small sample sizes. These ideas motivate a regression model whose coefficient matrix has low rank. Several approaches to reduced-rank regression have been developed to take advantage of this opportunity.

Consider again the linear regression model in Eq. (2). Here, ${\mathbf {B}}$ is a $P\times Q$ matrix of regression coefficients, where *P* is the number of covariates, and *Q* is the number of genes. In most gene expression studies, *Q* (and sometimes *P*) is large, and $\min (P, Q) \gg n$. The core assumption of reduced-rank regression (RRR) is that the matrix ${\mathbf {B}}$ has low rank [48]. In particular, the RRR model assumes ${\mathbf {B}}$ has rank $R \ll \min (P, Q)$. This implies that ${\mathbf {B}}$ can be factorized as an outer product of two low-rank matrices, giving us the following reduced-rank regression model:

3 $$\begin{aligned} {\mathbf {Y}} = {\mathbf {X}} {\mathbf {B}} + \varvec{\epsilon }~~~~\text {subject to } {\mathbf {B}} = {\mathbf {U}} {\mathbf {V}}^\top , \end{aligned}$$

where ${\mathbf {U}} \in {\mathbb {R}}^{P \times R}$ and ${\mathbf {V}} \in {\mathbb {R}}^{Q \times R}$. In the context of gene expression studies, this low-rank assumption implies that the relationship between cell-specific covariates and gene expression can be described in terms of a small set of latent factors. In other words, variance in gene expression is mediated by *R* different programs encoded in subsets of covariates; then ${\mathbf {B}}$ captures both the covariates of interest and their effect sizes within each of the *R* programs.

Several estimation approaches have been proposed for RRR under the assumption of Gaussian noise. A common method is to find the parameter values that minimize the squared reconstruction error [48, 49]:

$$\begin{aligned} \min _{{\mathbf {U}}, {\mathbf {V}}} \Vert {\mathbf {Y}} - {\mathbf {X}} {\mathbf {U}} {\mathbf {V}}^\top \Vert _2^2. \end{aligned}$$

This approach corresponds to finding the maximum likelihood solution of an RRR model with Gaussian errors ($\varvec{\epsilon }_q \sim {\mathcal {N}}(0, \sigma ^2 {\mathbf {I}})$ for $q = 1, \dots , Q$ in Eq. 3 as $\sigma ^2 \rightarrow 0$). When ${\mathbf {B}}$ is assumed to have full rank (that is, $R = \min (P, Q)$) the minimization admits the ordinary least squares (OLS) solution:

$$\begin{aligned} \widehat{{\mathbf {B}}}_{OLS} = ({\mathbf {X}}^\top {\mathbf {X}})^{-1} {\mathbf {X}}^\top {\mathbf {Y}}. \end{aligned}$$

When $R < \min (P, Q)$, the RRR model has an eigenvalue solution:

$$\begin{aligned} \widehat{{\mathbf {B}}}_{RRR} = \widehat{{\mathbf {B}}}_{OLS} {\mathbf {U}}_{1:R} {\mathbf {U}}_{1:R}^\top , \end{aligned}$$

where ${\mathbf {X}} \widehat{{\mathbf {B}}}_{OLS} = {\mathbf {U}}{\mathbf {D}}{\mathbf {V}}^\top$ is the SVD of the fitted values, and ${\mathbf {U}}_{1:R} = [{\mathbf {u}}_1, \cdots , {\mathbf {u}}_R]$ contains the leading *R* left singular vectors of ${\mathbf {X}} \widehat{{\mathbf {B}}}_{OLS}$.

Sparse approaches to RRR have been proposed as well. Sparsity in the decomposition leads to greater interpretability by including nonzero weights only on a subset of the covariates and genes for any component. One model [50] imposes sparsity on the coefficient matrix ${\mathbf {B}}$ by taking an iterative approach to estimation, solving both a sparse regression problem and the reduced-rank decomposition in alternating frames. The base algorithm solves the following optimization problem:

$$\begin{aligned} \min _{{\mathbf {U}}, {\mathbf {V}}} \frac{1}{2} \Vert {\mathbf {Y}}-{\mathbf {X}}{\mathbf {U}}{\mathbf {V}}^\top \Vert _2^2 + \lambda \sum ^{p}_{j=1}\Vert {\mathbf {U}}_j\Vert _2, \end{aligned}$$

where ${\mathbf {U}} \in {\mathbb {R}}^{P \times R}$, ${\mathbf {V}} \in {\mathbb {R}}^{Q \times R}$, ${\mathbf {U}}_p$ represents the *p*th row of ${\mathbf {U}}$, the rank *R* is specified by the modeler, and $\lambda$ is a sparsity penalty parameter. The alternating minimization problem can be broken into two steps: optimizing ${\mathbf {U}}$, and optimizing ${\mathbf {V}}$. After parameter initialization on iteration $\ell = 1$, on iteration $\ell = 2, \dots , L$, the algorithm first solves an orthogonal Procrustes problem for ${\mathbf {V}}$:

4 $$\begin{aligned} {\mathbf {V}}^{(\ell )} = \mathop {\mathrm {arg\,min}}\limits _{{\mathbf {V}}:{\mathbf {V}}{\mathbf {V}}^\top ={\mathbf {I}}} \Vert {\mathbf {Y}} - {\mathbf {X}}{\mathbf {U}}^{(\ell - 1)}{\mathbf {V}}^\top \Vert _2^2, \end{aligned}$$

where ${\mathbf {U}}^{(\ell - 1)}$ is the estimate of ${\mathbf {U}}$ from the previous iteration. The algorithm then solves a group lasso problem for ${\mathbf {U}}$:

5 $$\begin{aligned} {\mathbf {U}}^{\ell } = \mathop {\mathrm {arg\,min}}\limits _{{\mathbf {U}}} \frac{1}{2} \Vert {\mathbf {Y}}{\mathbf {V}}^{(\ell )}-{\mathbf {X}}{\mathbf {U}}\Vert _2^2 + \lambda \sum ^{P}_{p=1} \Vert {\mathbf {U}}_j\Vert _2. \end{aligned}$$

Equation (4) can be solved using a singular value decomposition, and Eq. (5) can be solved using techniques for group lasso [51]. These two steps are repeated for *L* steps or until convergence.

Another approach developed a Bayesian RRR framework for association mapping in the GWAS setting [52]. The model—called Bayesian Extendable Reduced-Rank Regression (BERRRI)—uses a nonparametric Indian Buffet Process prior for the latent factors, which allows the rank *k* to be estimated from the data. BERRRI then uses a variational Bayes approximation to the posterior for inference of the model parameters. However, BERRRI does not explicitly model count-based data, and its inference procedure is not computationally tractable for genome-scale analyses.

The linear RRR model has been generalized to nonlinear functions as well. The most popular nonlinear approaches have used neural networks with multiple inputs and multiple outputs [53]. The linear RRR model is equivalent to a single-layer multi-layer perceptron with only linear transformations between layers [54, 55]. This model can be extended to the nonlinear case by including nonlinear activation functions [54, 56]. However, these models typically to do not capture count data and lack the interpretability of linear models for downstream association testing.

In this manuscript, we propose a statistical model and associated computational framework that addresses the problems that arise with modeling genotype-phenotype associations for high-dimensional phenotypes captured with count data. We propose a reduced-rank regression model that finds low-dimensional associations between genotypes (or other high-dimensional covariates) and RNA-seq data (or other high-dimensional count-based phenotypes). Relying on low-dimensional associations alleviates the problem of estimating millions of pairwise associations. Furthermore, our model uses count-based likelihoods that allow both single-cell RNA-sequencing data and bulk RNA-sequencing data. We show that our approach appropriately models gene expression data with count-based likelihoods, leads to interpretable subsets of genes and genetic variants or other covariates in each dimension, and uses flexible, computationally tractable inference methods that allow for uncertainty quantification.

## Methods

We propose a probabilistic reduced-rank regression model with a Poisson data likelihood—which we call Poisson reduced-rank regression (PRRR)—for association mapping in count-based sequencing data. Our approach takes the form of a reduced-rank regression model with intermediate factors that explicitly model count-based data using a Poisson likelihood. These factors are interpretable and can be used to to identify and analyze the global structure of associations between cell covariates and cell phenotypes, such as gene expression levels. We ensure that inference is tractable and efficient in these models by using stochastic variational inference.

### Poisson reduced-rank regression (PRRR)

PRRR is designed to identify associations between cell-specific covariates and high-dimensional gene expression profiles. The response matrix ${\mathbf {Y}} \in {\mathbb {N}}_0^{N \times Q}$ is a matrix containing (in this application) RNA transcript counts for *Q* genes in *N* cells, where ${\mathbb {N}}_0 = {\mathbb {N}}\cup 0$. The $N \times P$ matrix ${\mathbf {X}}$ is a design matrix containing covariates for each cell. For example, these covariates could represent cell type, genotype, or measures of cell health.

PRRR uses a Poisson likelihood to model the transcript counts for each cell as the response variables, conditional on observed cell-specific covariates. The Poisson rate is parameterized by a low-rank linear mapping from the cell covariates.

Specifically, the transcript count of gene *p* in cell *n*, denoted by $y_{np}$ is modeled as a draw from a Poisson distribution, $y_{np} \sim \text {Poisson}(\lambda _{np})$. The Poisson rate $\lambda _{np}$ is determined by a linear function of the vector of covariates for cell *n*, denoted as ${\mathbf {x}}_n$. We use a canonical link function from the exponential family to map the domain of the latent variables to the positive real line—similar to a GLM approach. In particular, we use a $\log$ link function to ensure that, when pushed through the inverse link—the $\exp$ function—the mapped linear predictor, or the Poisson rate parameter, lies in ${\mathbb {R}}_+$. The likelihood model is then

6 $$\begin{aligned} y_{np} | {\mathbf {U}}, {\mathbf {V}}, {\mathbf {x}}_n \sim \text {Poisson} (\exp ({\mathbf {x}}_n {\mathbf {U}}{\mathbf {v}}_{p\cdot }^\top )), \end{aligned}$$

where ${\mathbf {v}}_{p\cdot }$ is the *p*th row of ${\mathbf {V}}$. We place Gaussian priors on columns of ${\mathbf {U}}$ and ${\mathbf {V}}$:

7 $$\begin{aligned} {\mathbf {u}}_{r} \sim {\mathcal {N}}({\mathbf {0}}, \sigma ^2_1 {\mathbf {I}}_P),~~~{\mathbf {v}}_{r} \sim {\mathcal {N}}({\mathbf {0}}, \sigma ^2_2 {\mathbf {I}}_Q), \end{aligned}$$

for $R = 1, \dots , R$. Intuitively, ${\mathbf {U}}$ and ${\mathbf {V}}$ capture the low-rank associations between ${\mathbf {X}}$ and ${\mathbf {Y}}$.

#### Nonnegative PRRR

In some cases, the covariates ${\mathbf {X}}$ are entirely nonnegative — possibly representing counts or categories—in which case it may be of interest to identify nonnegative, low-rank regression coefficients that explain the associations in a completely additive fashion. For example, in eQTL mapping, the covariates are typically the count of the minor allele for each SNP, where ${\mathbf {x}}_n \in \{0, 1, 2\}$, and it may be of interest to identify a nonnegative, “parts-based” combination of SNPs that explain phenotypic variation. For these cases, we propose nonnegative Poisson reduced-rank regression (nn-PRRR), whose likelihood is given by

8 $$\begin{aligned} y_{np} | {\mathbf {U}}, {\mathbf {V}}, {\mathbf {x}}_n \sim \text {Poisson} (s_n {\mathbf {x}}_n^\top {\mathbf {U}} {\mathbf {v}}_{p\cdot }^\top ), \end{aligned}$$

where $s_n$ is a cell-specific size factor modeling the total number of transcripts in cell *i*. We fix $s_n$ to be the total number of transcript counts in cell, $s_n = \sum _{j=1}^p y_{np}$. We place Gamma priors on the elements of ${\mathbf {U}}$ and ${\mathbf {V}}$:

9 $$\begin{aligned} u_{pr} \sim \text {Gamma}(\alpha _u, \beta _u),~~~v_{qr} \sim \text {Gamma}(\alpha _v, \beta _v) \end{aligned}$$

for $p = 1,\dots ,P$, $q = 1, \dots , Q$, and $r = 1, \dots , R$. For all experiments, we set $\alpha _u=\alpha _v=2$ and $\beta _u=\beta _v=1$. Thus, the structures of the PRRR and nn-PRRR models are similar except for the nonnegativity constraint and associated prior (Additional file 1: Fig. S1).

#### Choosing between PRRR and nn-PRRR

While the PRRR and nn-PRRR models are designed for the same goal — performing reduced-rank regression with high-dimensional, count-based outcomes—some care is required when choosing which model to apply for a particular application. The choice largely depends on the goal of the analysis. If a primary goal is to examine the low-dimensional latent factors in a dataset, then nn-PRRR is often preferable because its nonnegative factors encourage a parts-based representation, which may be easier to interpret. nn-PRRR also requires that the covariates are nonnegative. On the other hand, when the primary goal is prediction, PRRR may be preferable because its real-valued factors will be less constrained due to the removal of the nonnegativity constraint. However, these are only guidelines and not hard restrictions, and often it may be preferable to fit both PRRR and nn-PRRR to a dataset, when possible, and study both sets of results to select the one with the more appropriate behavior.

### Estimation and inference

We propose two approaches to fit our model to data: 1) computing a point estimate for the coefficients using maximum *a posteriori* (MAP) methods and 2) full Bayesian posterior inference for the regression coefficients using an approximate inference procedure.

#### MAP estimation

The MAP solution in our model is the coefficient matrices ${\mathbf {U}}_{MAP}, {\mathbf {V}}_{MAP}$ with maximum posterior probability given the data ${\mathbf {X}}$ and ${\mathbf {Y}}$. In particular,

$$\begin{aligned} {\mathbf {U}}_{MAP}, {\mathbf {V}}_{MAP} = \mathop {\mathrm {arg\,max}}\limits _{{\mathbf {U}}, {\mathbf {V}}} p({\mathbf {U}}, {\mathbf {V}} | {\mathbf {X}}, {\mathbf {Y}}). \end{aligned}$$

Expanding the posterior with Bayes’ rule, we can write the MAP objective as $\max _{{\mathbf {U}}, {\mathbf {V}}} p({\mathbf {Y}} | {\mathbf {X}}, {\mathbf {U}}, {\mathbf {V}}) p({\mathbf {U}}, {\mathbf {V}}) / Z,$ where *Z* is a normalizing constant that does not depend on ${\mathbf {U}}$ or ${\mathbf {V}}$. Taking a $\log$, dropping the constant *Z*, and leveraging the i.i.d. assumption, we arrive at our final objective for the MAP estimate of the model parameters:

$$\begin{aligned} {\mathbf {U}}_{MAP}, {\mathbf {V}}_{MAP} = \mathop {\mathrm {arg\,max}}\limits _{{\mathbf {U}}, {\mathbf {V}}} \log p({\mathbf {U}}, {\mathbf {V}}) + \sum \limits _{n=1}^N \log p({\mathbf {y}}_n | {\mathbf {x}}_n, {\mathbf {U}}, {\mathbf {V}}). \end{aligned}$$

Although this maximization problem does not have a closed-form solution, we use gradient-based methods to iteratively maximize this log posterior with respect to ${\mathbf {U}}$ and ${\mathbf {V}}$.

### Variational inference

A fully Bayesian approach to inference, given a set of samples with paired cell covariates and transcript counts, $\{({\mathbf {x}}_n, {\mathbf {y}}_n)\}_{n=1}^N$, would compute the posterior distribution of the parameters, ${\mathbf {U}}$ and ${\mathbf {V}}$, given the data matrices ${\mathbf {X}}$ and ${\mathbf {Y}}$. By Bayes’ rule,

$$\begin{aligned} p({\mathbf {U}}, {\mathbf {V}} | {\mathbf {X}}, {\mathbf {Y}}) = \frac{ p({\mathbf {X}}, {\mathbf {Y}} | {\mathbf {U}}, {\mathbf {V}}) p({\mathbf {U}}, {\mathbf {V}})}{p({\mathbf {X}}, {\mathbf {Y}})}. \end{aligned}$$

However, the marginal likelihood, $p({\mathbf {X}}, {\mathbf {Y}})$, contains an intractable integral,

10 $$\begin{aligned} p({\mathbf {X}}, {\mathbf {Y}})=\int _{{\mathcal {U}} \times {\mathcal {V}}} p({\mathbf {X}}, {\mathbf {Y}}, {\mathbf {U}}, {\mathbf {V}}) d{\mathbf {U}}d{\mathbf {V}}. \end{aligned}$$

To circumvent this issue, we use a variational approximation to the posterior. Specifically, we use a mean-field variational approximation, $p({\mathbf {U}}, {\mathbf {V}}) \approx q({\mathbf {U}}, {\mathbf {V}}) = q_1({\mathbf {U}})q_2({\mathbf {V}})$, where $q_1$ and $q_2$ are the variational distributions. Here, we specify the variational families for PRRR and nn-PRRR to be normal and log normal, respectively,

11 $$\begin{aligned} q(u_{pr})&= {\mathcal {N}}(\mu _1, \sigma ^2_1),~~~~~~~~q(v_{qr}) = {\mathcal {N}}(\mu _2, \sigma ^2_2),&\text {(PRRR)} \end{aligned}$$

12 $$\begin{aligned} q(u_{pr})&= \text {Log} {\mathcal {N}} (\mu _1, \sigma ^2_1),~~~q(v_{qr}) = \text {Log} {\mathcal {N}} (\mu _2, \sigma ^2_2).&\text {(nn-PRRR)} \end{aligned}$$

We minimize the KL divergence between the true posterior and the variational approximation, which is equivalent to maximizing a lower bound on the model evidence (called the ELBO). This lower bound for PRRR is given by

$$\begin{aligned} p({\mathbf {X}}, {\mathbf {Y}}) \ge {\mathcal {L}} := {\mathbb {E}}_{q({\mathbf {U}})q({\mathbf {V}})}\left[ \frac{p({\mathbf {X}}, {\mathbf {Y}}, {\mathbf {U}}, {\mathbf {V}})}{q({\mathbf {U}})q({\mathbf {V}})}\right]. \end{aligned}$$

We maximize this lower bound with respect to the variational parameters using stochastic variational inference [57] as implemented in TensorFlow Probability [58]. For all experiments, we use the Adam optimizer [59] with a learning rate of 0.01.

## Results

### Simulation experiments

We first demonstrate the use cases of PRRR and test the robustness and accuracy of our model using simulated data.

#### PRRR identifies low-dimensional association maps

We first sought to confirm that PRRR identifies the low-dimensional relationships between covariates and outcomes.

**Fig. 1:**
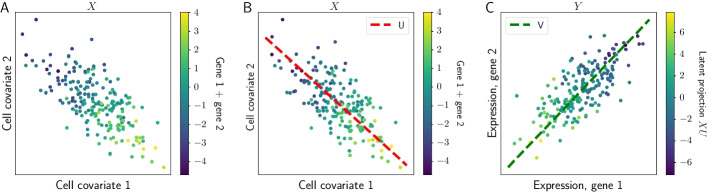
Illustration of PRRR. We fit PRRR to a toy dataset containing two cell-specific covariates and two genes. The two covariates showed negative correlation, and the two genes were jointly associated with the covariates (panel **A**). PRRR identifies the low-rank structure of these multivariate relationships by decomposing the full coefficient matrix into two low-rank matrices, *U* and *V* (panels **B** and **C**)

To start in a setting that can be visualized, we generated a synthetic dataset in which the covariates and outcomes are both two-dimensional. Specifically, we sampled data from the generative model (Eqs. 8, 9), setting $R=1$. We forced a correlation between the covariates and outcomes. We found that PRRR could reliably detect the one-dimensional association between ${\mathbf {X}}$ and ${\mathbf {Y}}$ (Fig. 1). Moreover, we are able to recover a quantification of the relationship between the covariates and outcomes, and visualize this relationship in the low-dimensional space.

**Fig. 2:**
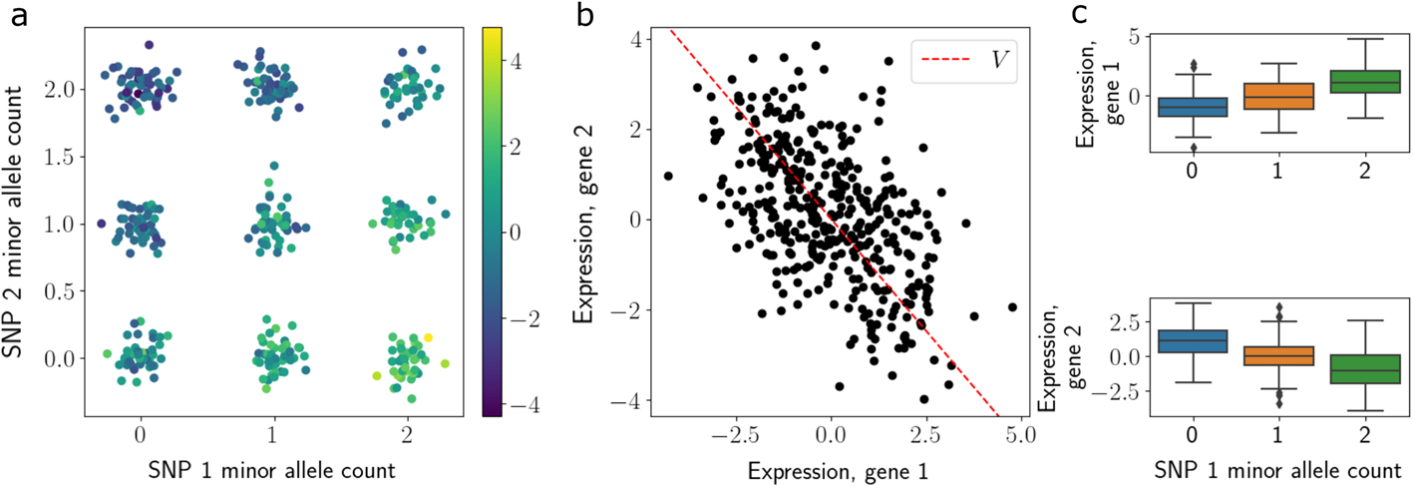
eQTL mapping in simulated single-cell data with PRRR. Toy example demonstrating eQTL mapping with PRRR for two genetic variants and two genes. **a** Genotype data, shown as the number of copies of the minor allele for variant 1 (x-axis) and variant 2 (y-axis) and colored by each sample’s corresponding expression of gene 1. **b** Gene expression values. The red line represents the fitted value for $\mathbf {V}$ with $r=1$ in this toy example for gene 1 (x-axis) and gene 2 (y-axis). **c** Relationship between genotype (x-axis) and gene expression (y-axis) for the two genes

We next extended this simulation study and visualization to a small-scale synthetic eQTL study. We generated $N = 200$ synthetic genotypes based on minor allele counts, ${\mathbf {x}}_n \in \{0, 1, 2\}$, and sampled synthetic RNA transcript counts using the PRRR generative model, with $P=Q=2$ for visualization. We fit PRRR to these data and inspected the fitted coefficients. We found that PRRR recovered these genotype-expression relationships, and allowed for both inspection of the low-dimensional structure of these relationships, as well as investigating univariate relationships (Fig. 2). This experiment suggests that PRRR may be useful to perform eQTL mapping.

#### PRRR identifies the optimal rank and is robust to misspecification

PRRR, like other reduced-rank regression approaches, requires selecting the rank *R* of the coefficient matrix. A common approach is to evaluate the goodness-of-fit of the model at varying values of *R*, and select the one with the best fit to the data. To test whether this is feasible with PRRR, we use synthetic data that was generated from PRRR’s generative model with true rank $R^\star =3$. We then fit the model with $R \in \{1, 2, \dots , 10\}$ and compute the ELBO for each fit. We repeated this experiment 30 times for each value of *R*.

**Fig. 3:**
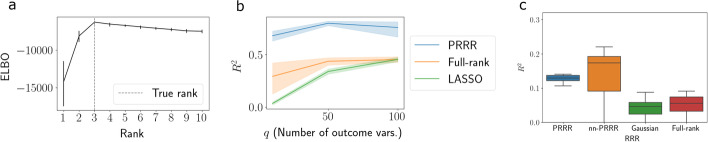
PRRR identifies optimal rank and is robust to data dimension. **a** Using synthetic data generated under the PRRR model with a true rank of $R^\star = 3$, we fit PRRR with a range of rank specifications on the x-axis, where $R^{\star } = 3$. The y-axis shows the ELBO values for each model rank. Vertical ticks represent $95\%$ confidence intervals. **b** Goodness-of-fit $R^2$ values for predictions from PRRR, a full-rank version of PRRR, and a multi-output LASSO model [51] for outcome data with dimension $Q \in \{10, 50, 100\}$. **c** Goodness-of-fit $R^2$ values for predictions from PRRR, nn-PRRR, and competing models for outcome data generated from Splatter [60]

We find that the PRRR ELBO peaked at the true value of $R=3$ (Fig. 3a), demonstrating that the model’s fit to the data was best at the true rank $R^{\star }$. Moreover, we found that, while the goodness-of-fit sharply degraded for models with $R < R^\star$, the goodness-of-fit declined more slowly for models with $R > R^\star$. This finding confirms similar observations from previous studies [50], and suggests that setting the rank to be higher than anticipated is protective against model misspecification.

#### PRRR is robust to data dimension

We next sought to validate the robustness of PRRR in the presence of higher-dimensional data. To do so, we generated three datasets from the PRRR model, each with a different number of response features (genes), $Q \in \{10, 50, 100\}$. We set the sample size to $N = 200$, and we randomly selected $80\%$ of these samples to fit the model and held out the remaining $20\%$ to test the model. We fit PRRR on the training data using our MAP estimation procedure and used the estimated parameters to compute the predicted Poisson rate for the held-out data, $\widehat{\lambda }_{np} = \exp ({\mathbf {x}}_n \widehat{{\mathbf {U}}}\widehat{{\mathbf {v}}}_{p\cdot }^\top$. We then computed the goodness-of-fit $R^2$ measure between our predictions and the held-out dataset’s counts. To benchmark these predictions, we compared PRRR’s predictive performance to two competing methods: a full-rank version of PRRR and a multi-output LASSO model, as implemented in the R package glmnet [51]. We repeated this experiment ten times for each method and each data-generating condition.

We found that PRRR reliably achieves good predictive performance across all values of the outcome dimension *Q* (Fig. 3b). In contrast, the full-rank and LASSO models performed worse.

To further validate PRRR and nn-PRRR on different data types, we conducted a similar experiment with synthetic data generated using Splatter [60], a data simulator designed specifically for single-cell count data. We generated data for $N=200$ samples, each belonging to one of 10 groups, and used $Q=100$ genes. We used the one-hot encoded group labels as the covariates and the synthetic gene expression as the response. We fit PRRR and nn-PRRR with $R=5$, along with Gaussian RRR and full-rank Poisson regression, and we reserved a hold-out set for evaluating predictions. We found that PRRR and nn-PRRR outperformed competing models in terms of their predictive $R^2$ values (Fig. 3c).

These results suggest that accounting for the count-based data and the low-rank structure of associations is vital, and that the PRRR model successfully captures this structure.

#### PRRR predictions are robust to rank misspecification

To further explore the role of rank specification in our model, we performed a prediction experiment for varying settings of the rank. We generated synthetic data as before with $R^\star = 3$ and fit the model on $80\%$ of the data while reserving $20\%$ for testing. For a range of ranks, $R \in \{1, 2, 3, 4, 5, 10, 20\}$, we fit PRRR, used the fitted model to make predictions for the held-out data, and computed the $R^2$ coefficient of determination for these predictions. We performed this experiment using both maximum a posteriori (MAP) estimation and variational inference to fit the model, repeating the experiment ten times for each rank in both cases.

**Fig. 4:**
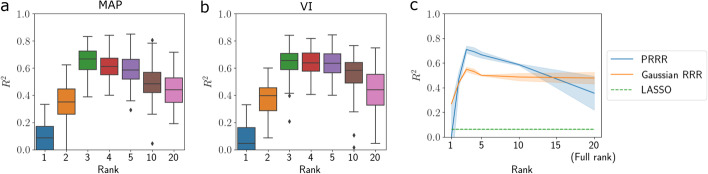
PRRR is robust to rank misspecification. Using synthetic data generated from the PRRR model with a true rank of $R^\star = 3$, we fit PRRR with a range of rank specifications (*x*-axis). We made predictions for a held-out dataset and computed the $R^2$ coefficient of determination, repeating this ten times for each rank. The *y*-axis shows the $R^2$ value between the predicted values and the true values on held-out samples. Boxes show the median and upper and lower quartiles, and whiskers extend to 1.5 times the interquartile range. **a** Maximum *a posteriori* estimates (MAP); **b** Variational inference (VI); **c** Comparison with Gaussian RRR [48] and LASSO [51]

We found that the predictive performance was strongest when the model was correctly specified ($R = 3$ in this case; Fig. 4a, b). However, we observed that performance was strong across a range of misspecified ranks as well. Similar to our previous experiment, we observed that predictions were more robust for models with $R > R^\star$ as compared to models with $R < R^\star$.

To benchmark these predictions, we compared PRRR’s predictive performance to two competing methods: a reduced-rank regression that assumes a Gaussian likelihood [48] and the multi-output LASSO model [51]. We performed a similar prediction experiment as above, computing the $R^2$ for each model under a range of rank specifications. We found that PRRR outperformed the two competing methods in a range around the true rank $R^\star = 3$ (Fig. 4c).

### Characterizing transcriptional hallmarks of pancreatic cell types

It has been widely observed that cell type is a major driver of transcriptional variation between cells in a variety of tissue types [4, 61–63]. Given these observed differences between cell types, it is of interest to identify the gene expression patterns that are characteristic of each cell type. Our PRRR models present a principled approach for identifying these transcriptional hallmarks of cell type by finding a low-dimensional mapping from cell type to expression.

To test this, we fit PRRR to an scRNA-seq dataset containing 1,578 cells that span $C=14$ unique cell types in the human pancreas [64]. For cell *n*, we encode its cell type as a one-hot vector ${\mathbf {x}}_n \in \{0, 1\}^{C}$, and we treat the response variable ${\mathbf {y}}_n$ as the vector of RNA transcript counts in this cell. We extracted the coefficient matrices ${\mathbf {U}}$ and ${\mathbf {V}}$ and studied their properties. For comparison, we also fit BERRRI [52] and a multi-layer, multi-output neural network [54, 55]. For BERRRI, we set an upper limit of 13 latent factors, and set the rest of the parameters to their default settings. For the neural network, we included two hidden layers and used the Adam optimizer [59].

We found that PRRR was able to identify transcriptional markers in each cell type. Among the 14 unique cell types present in the dataset, there are five that belong to the family of islet cells (*alpha*, *beta*, *gamma*, *delta*, and *epsilon* cells). Given their functional relatedness, these cell types are expected to show similar gene expression patterns compared to patterns found in other cell types. The competing methods, BERRRI and a neural network, were unable to separate islet and non-islet cell types in their latent spaces (Additional file 2: Fig. S2).

**Fig. 5:**
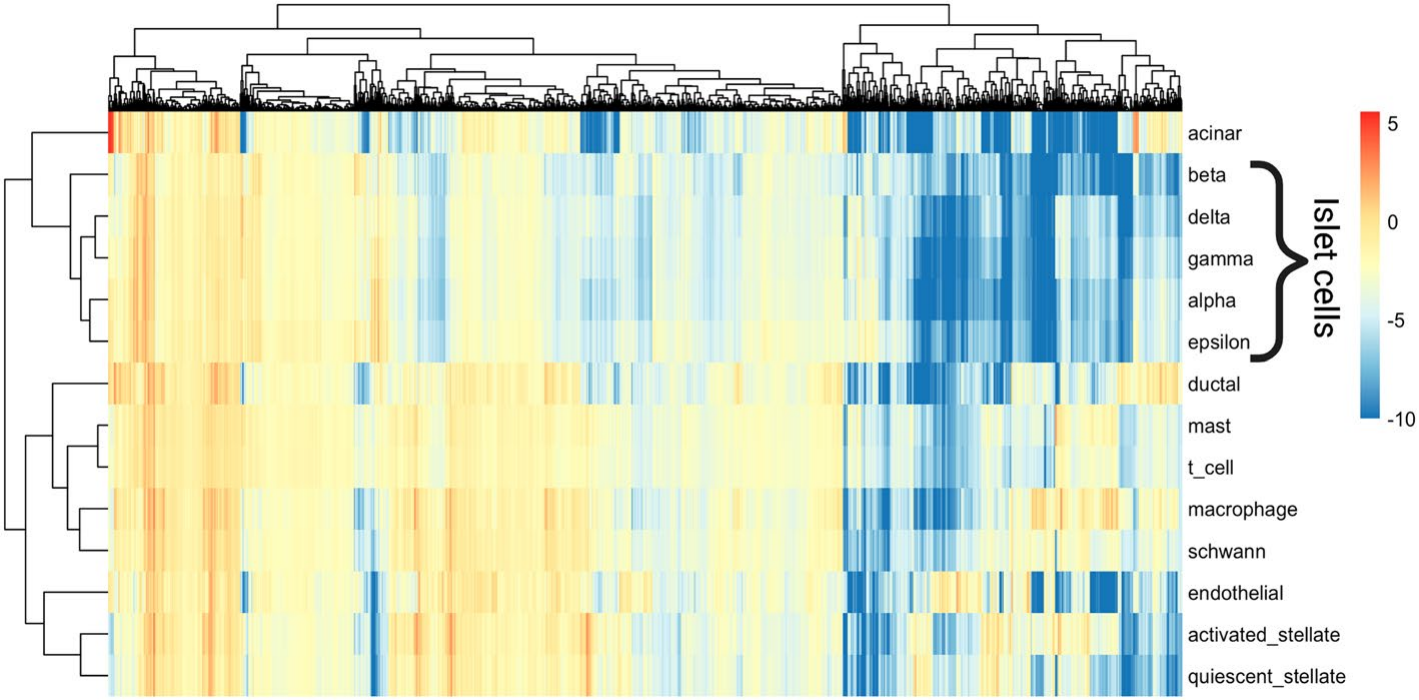
PRRR coefficients for pancreatic cell types. Heatmap showing the full coefficient matrix $\mathbf {U} \mathbf {V}^\top$, with cell types on the rows and genes on the columns

**Fig. 6:**
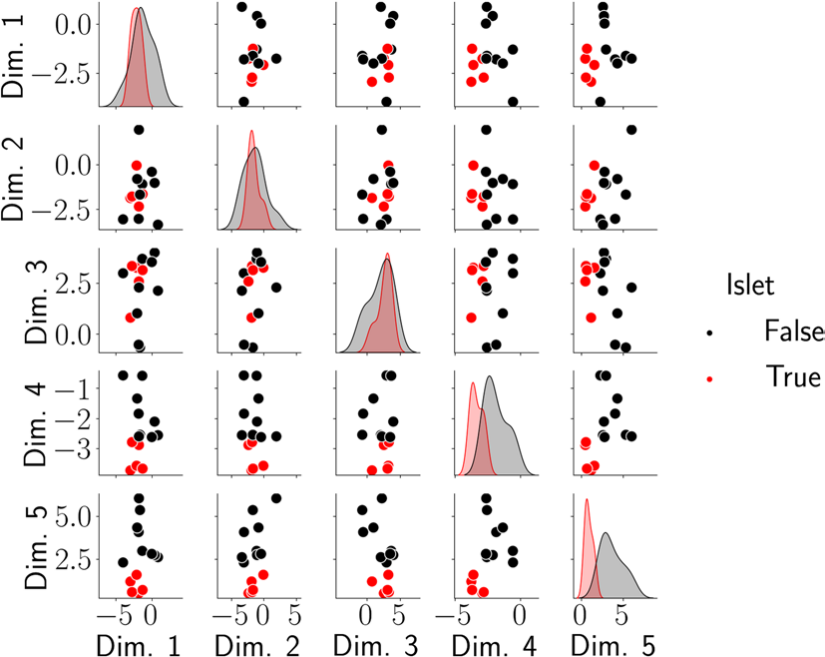
PRRR identifies similar expression patterns in islet cell types. Shown here is the latent encoding of each cell type for each pair of latent variables in $\mathbf {U}$, where PRRR was fitted with $R=5$ Each point in each subplot represents a cell type, and cell types are colored by whether they are classified as islet cells or not. The densities on the diagonal show the distribution of $\mathbf {U}$ values for islet and non-islet cell types in each latent dimension

**Fig. 7:**
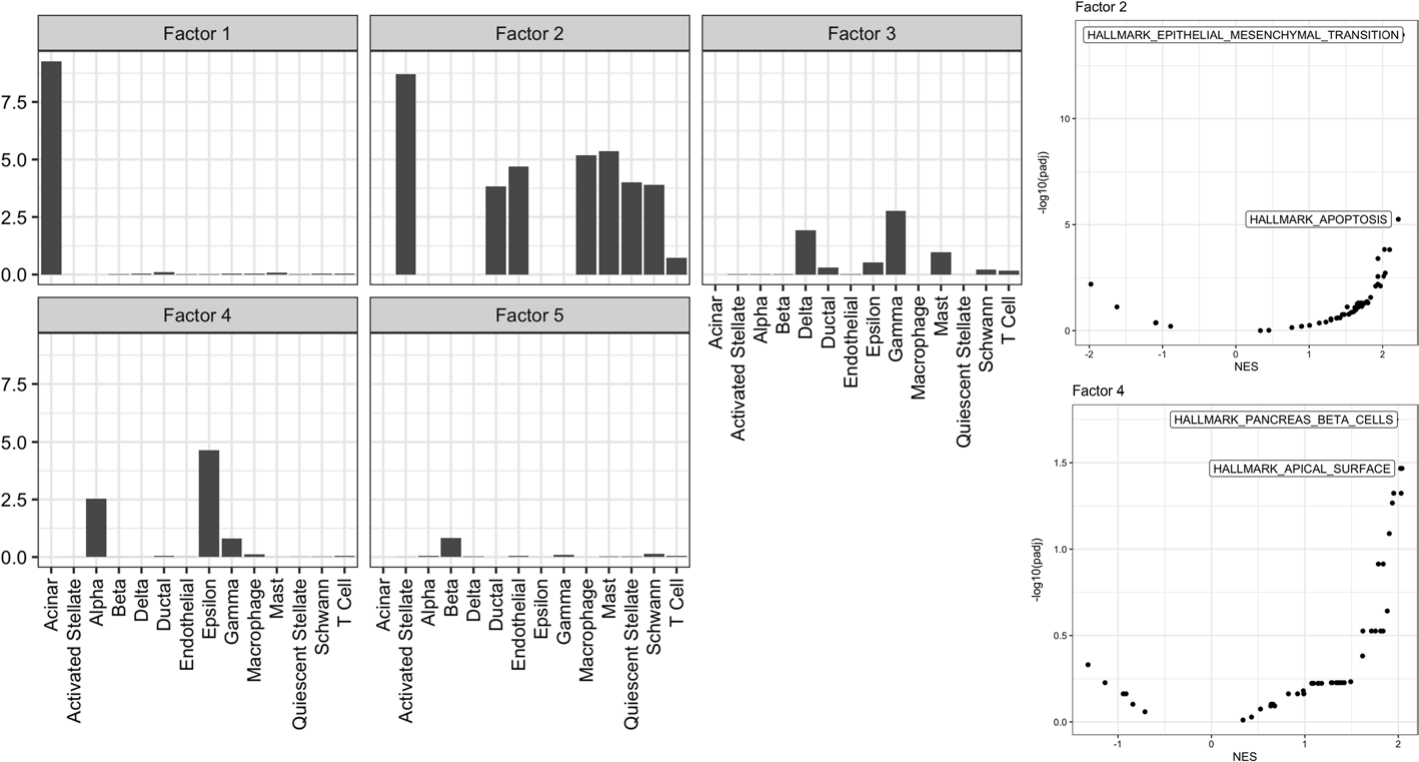
PRRR factors identify subgroups of cell types. **a** shows each cell type’s loading onto each of the five latent factors in the $\mathbf {U}$ matrix. **b** shows a gene set enrichment analysis of the gene loadings onto factors 2 and 4 in the $\mathbf {V}$ matrix

Indeed, inspecting PRRR’s estimated coefficients, we find that the model captures the low-dimensional gene expression patterns in islet cells (Fig. 5). We performed a hierarchical clustering on the PRRR coefficients, which revealed that the islet cells clustered together (Fig. 5). We found a similar clustering after fitting nn-PRRR on the same dataset (Additional file 3: Fig. S3). Moreover, we observed that the models separated islet cell types from non-islet cell types in the low-dimensional space (Fig. 6). Examining the factor loadings for each cell type, we found that specific factors were especially enriched for islet or non-islet cell types (Fig. 7). The islet-related factors were enriched for pancreatic gene pathways, such as *pancreas beta cells*.

We also extracted the top genes for each cell type from the full PRRR coefficient matrix; these genes can be viewed as “marker genes” whose expression is correlated with certain cell type identities. We found that these marker genes correspond to cell-type-specific transcription patterns, such as *REG1B* being the top marker gene for *acinar* cells (Additional file 4: Fig. S4, Additional file 5: Fig. S5).

These findings imply that the low-dimensional space may be used to compare and contrast existing classifications within the data, and to discover possible new relationships among covariates and phenotypes. They also show that PRRR is able to identify the distinct transcriptional characteristics of specific cell types and groups of cell types, and that PRRR could be used to identify marker genes for cell types.

### Analyzing gene expression patterns in spatial datasets

Along with cell type, the physical organization of cells within a tissue has a strong impact on gene expression due to tissue organization structure and cell-cell communication. The rise of spatial gene expression profiling technologies provides an opportunity to study how gene expression levels vary spatially across a tissue [65–68]. In particular, given gene expression data at the individual cell level with appropriate spatial context, it is of interest to identify how the expression of specific genes varies across the expanse of a tissue.

To study this with our modeling framework, we fit PRRR with rank $R=1$ to a two-dimensional spatial dataset containing 2,063 mouse brain sagittal anterior cells [69]. The ${\mathbf {X}}$ matrix is an $N \times 2$ matrix containing two-dimensional spatial coordinates for each cell *n*, and we treat the response variable ${\mathbf {Y}}$ as the matrix of RNA transcript counts. After fitting PRRR, we extracted the model coefficients to inspect the spatial trends in gene expression that it identified.

**Fig. 8:**
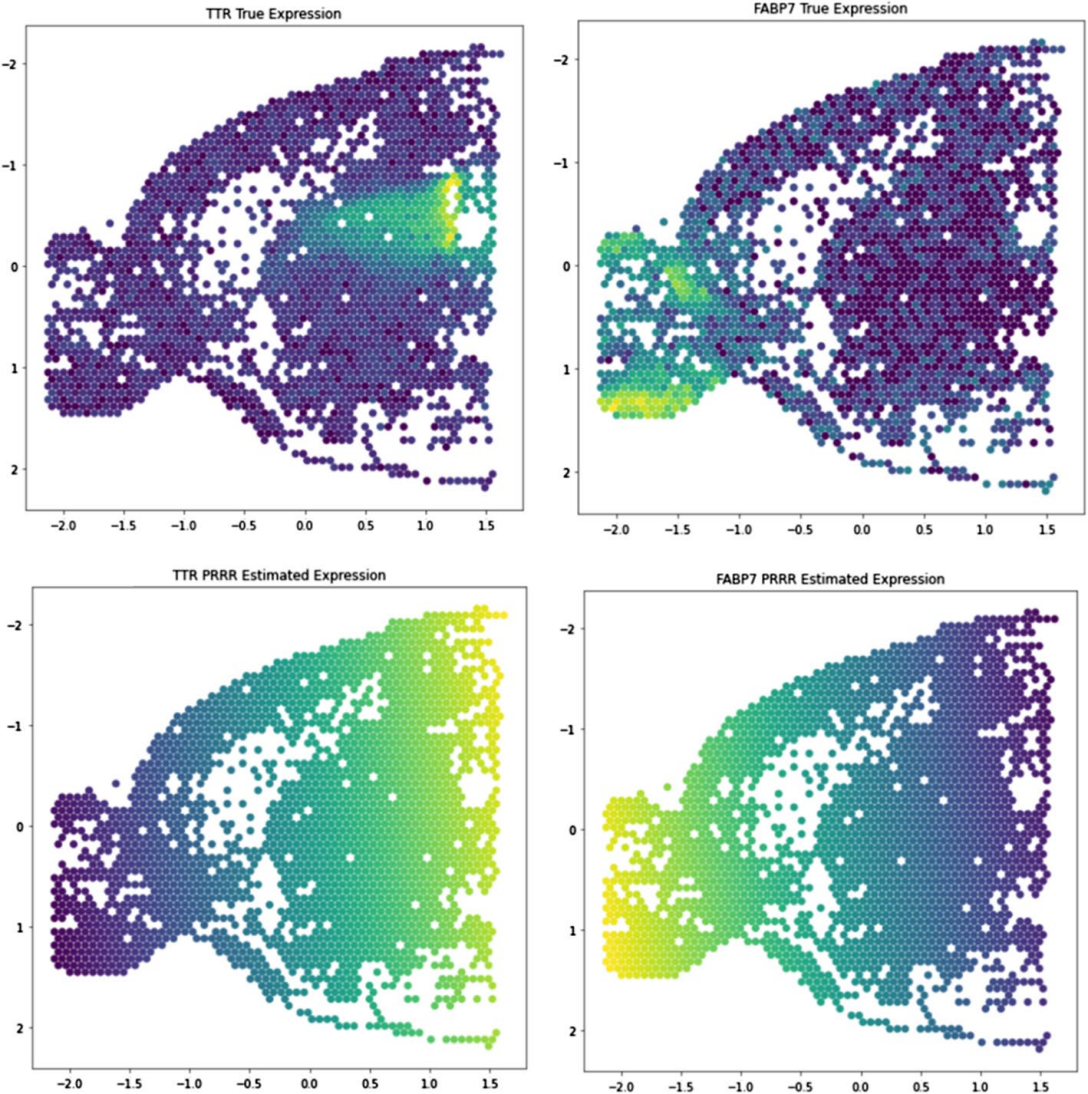
PRRR identifies directional patterns in spatial gene expression data. We applied PRRR to a Visium spatial gene expression readout from the sagittal anterior region of a mouse brain, using each spot’s spatial coordinates as the covariates and each spot’s gene expression levels as the outcome. Left: Spatial gene expression data for the gene *TTR* and PRRR’s estimated spatial pattern for this gene. Right: Spatial gene expression data for the gene *FABP7* and PRRR’s estimated spatial pattern for this gene

We found that PRRR is able to identify trends in gene expression along one latent dimension. While the model is constrained to only identify linear changes in gene expression across space, it is able to identify a general trend in increased gene expression for individual genes such as *TTR* and *FABP7* (Fig.  8). This result demonstrates the utility of our model in the context of spatial genomics and further demonstrates the versatility of PRRR.

### eQTL mapping

eQTL mapping is a common approach to finding associations between genotypes and gene expression profiles. However, this type of association mapping requires fitting a regression between millions of genotype variants and the expression of tens of thousands of genes, resulting in billions of univariate models [18]. The large number of univariate tests can cause these approaches to be prohibitive computationally and to lack sufficient statistical power.

We hypothesized that our reduced-rank regression model could alleviate these issues of computational tractability and statistical power. To test this, we applied PRRR to an eQTL mapping setting to find a set of low-dimensional factors that capture the relationships between genotype and gene expression. To do so, we used data from the Genotype Tissue Expression (GTEx) Consortium [19]. For this experiment, we focused on data collected from liver tissues from 227 donors. For each donor, the data consist of paired genotype (as encoded by minor allele count ${\mathbf {x}}_n \in \{0, 1, 2\}$) and bulk gene expression profiles. For our analysis, we used genotype data from chromosome 1 ($p=18,892$) and expression levels for the top $q=5,000$ most variable genes.

**Fig. 9:**
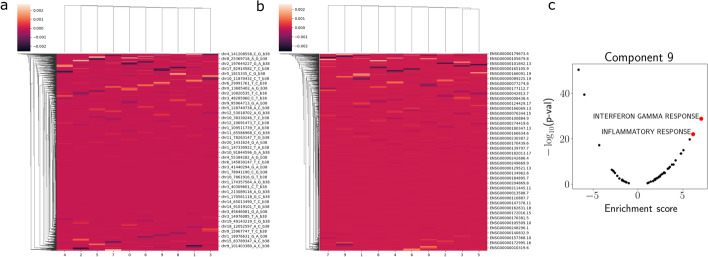
PRRR loadings matrices for the GTEx eQTL experiment. **a** A heatmap representation of the matrix $\mathbf {U},$ showing SNPs on the rows and latent dimensions on the columns. **b** A heatmap representation of the matrix $\mathbf {V},$ showing genes on the rows and latent dimensions on the columns. **c** Gene set enrichment analysis (GSEA) of component 9 in $\mathbf {V}$

**Table 1:** Gene set enrichment results for GTEx eQTL experiment

Factor	Pathway	Adjusted p-val	NES	Leading edge
1	ALLOGRAFT REJECTION	7.16e-05	1.15e+00	*FLNA*,*KRT1*,*RPS3A*
1	APICAL JUNCTION	1.13e-03	1.13e+00	*ACTB*,*MYL9*,*ACTG1*
1	EMT	5.32e-07	1.18e+00	*FN1*,*FLNA*,*TAGLN*
1	HYPOXIA	1.29e-03	1.12e+00	*DCN*,*ANXA2*,*FOS*
1	MYC TARGETS V1	1.80e-03	1.12e+00	*RPLP0*,*RPS6*,*RPS2*
1	MYOGENESIS	3.91e-06	1.17e+00	*MYH11*,*TAGLN*,*GSN*
1	P53 PATHWAY	2.61e-03	1.12e+00	*PERP*,*RACK1*,*TXNIP*
1	TNFA SIG. VIA NFKB	6.57e-03	1.11e+00	*FOS*,*DUSP1*,*CD44*
7	APICAL JUNCTION	1.79e-05	1.20e+00	*MYL9*,*MYH9*,*ACTB*
7	APOPTOSIS	8.01e-06	1.23e+00	*DCN*,*GPX3*,*GSN*
7	COAGULATION	3.60e-07	1.27e+00	*A2M*,*FN1*,*SPARC*
7	COMPLEMENT	3.83e-04	1.17e+00	*FN1*,*CSRP1*,*CLU*
7	EMT	7.22e-12	1.29e+00	*ACTA2*,*TAGLN*,*FN1*
7	HYPOXIA	2.62e-05	1.19e+00	*MYH9*,*DCN*,*BGN*
7	IL2 STAT5 SIGNALING	7.48e-03	1.13e+00	*COL6A1*,*AHNAK*,*TGM2*
7	MITOTIC SPINDLE	1.39e-03	1.15e+00	*FLNA*,*MYH9*,*GSN*
7	MYC TARGETS V1	1.20e-03	1.15e+00	*HSP90AB1*,*RPS6*,*RPLP0*
7	MYOGENESIS	2.45e-11	1.29e+00	*TAGLN*,*MYH11*,*COL6A2*
7	TNFA SIG. VIA NFKB	1.39e-03	1.15e+00	*NR4A1*,*RHOB*,*ZFP36*
7	UV RESPONSE DN	1.91e-03	1.17e+00	*COL1A2*,*IGFBP5*,*COL3A1*

*EMT* stands for epithelial mesenchymal transition

**Fig. 10:**
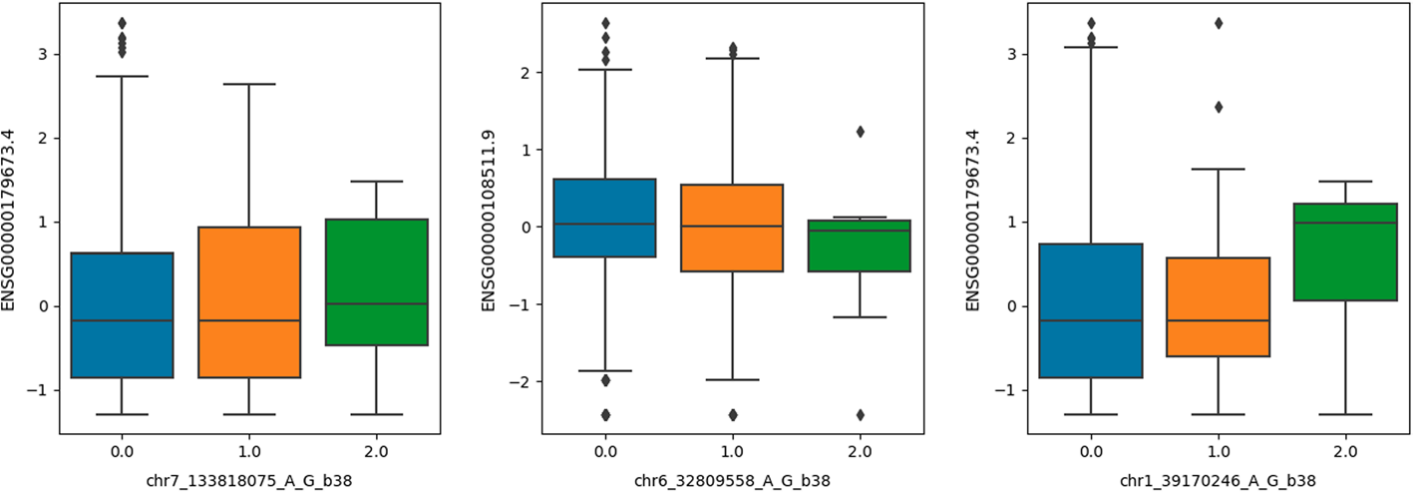
Three eQTL associations found in one latent factor of PRRR applied to GTEx liver samples

We fit PRRR with $R=10$ latent dimensions and extracted the low-rank regression coefficient matrix (Fig. 9a, b). Within each factor, we can examine associations between individual genetic variants and the expression of individual genes. For factor number *r*, we do this by taking the outer product of the corresponding columns of ${\mathbf {U}}$ and ${\mathbf {V}}$, respectively. That is, we compute ${\mathbf {u}}_r {\mathbf {v}}_r^\top$ and examine the strongest SNP-gene relationships (Fig. 10). Investigating the latent factors more closely, we found several meaningful associations. For example, after performing a gene set enrichment analysis (Table 1), we found that the gene expression loadings for factor 9 were enriched for genes related to *interferon gamma response* and *inflammatory response* (Fig. 9c), two major functional roles of liver cells [70, 71]. We find similar results for nn-PRRR, although the ${\mathbf {V}}$ matrix is much more sparse (Additional file 6: Fig. S6). The additional sparsity in the nn-PRRR results aligns well with the parts-based representation of the low-dimensional space known with nonnegative matrix factorizations [72–74]. This experiment suggests that the PRRR and nn-RRR framework may be useful for studying associations between genotypes and phenotypes, especially when there is low-dimensional correlation structure between the datasets and within each dataset individually.

## Discussion

In this paper, we present two reduced-rank regression models and associated variational inference approaches—Poisson RRR (PRRR) and nonnegative Poisson RRR (nn-PRRR)—to jointly model associations within two high-dimensional paired sets of features where the response variables are counts. In simulations, PRRR and nn-PRRR are able to effectively capture associations between paired high-dimensional data. Moreover, we show that these models can identify the optimal rank for the parameter matrix. In the context of sequencing data, we find that PRRR and nn-PRRR may be used for robust identification of cell types, quantifying the relationships between cell types, and performing association mapping of genetic variants to correlated genes.

There are several limitations of our proposed model. First, PRRR and nn-PRRR only model linear associations between inputs and outputs and are unable to account nonlinear relationships. Second, we observe that the time complexity for fitting PRRR and nn-PRRR is somewhat slower than competing models (Additional file 7: Fig. S7), although the fitting time is not prohibitive. Third, a limitation of our variational inference (and any variational inference) is that the variational posterior distribution is an approximation to the full posterior and not the exact posterior. Finally, our model requires the user to specify the number of latent dimensions, which may be difficult in practice; often the user will run the method with different latent dimension values and use the results that are the most interpretable (Additional file 8).

Several extensions of the model could be considered. A nonparametric prior could allow for flexibly learning the rank of the parameter matrix, rather than requiring the rank to be pre-specified, as in related work [52]. Additionally, the generalized model could be extended to different likelihood distributions. Furthermore, additional structure could be added to the latent variables, such as sparsity or a gene network [75, 76], to encode additional known structure in the covariates.

## Conclusions

We present a Poisson reduced-rank regression (PRRR) model, along with a nonnegative counterpart called nn-PRRR, for association mapping in count-based sequencing data. PRRR is able to detect associations between a high-dimensional response matrix and a high-dimensional set of predictors by leveraging low-dimensional representations of the data. Using principled Bayesian modeling, PRRR is able to properly account for the count-based nature of RNA sequencing data using a Poisson likelihood. We ensure that inference is tractable and efficient in these models by applying a fast variational inference approach.

## Supplementary Information

**Additional file 1**. **Fig. S1** Graphical model for PRRR and nn-PRRR.

**Additional file 2**. **Fig. S2** BERRRI and a neural network approach fail to identify expression patterns in islet celltypes. Shown here is the latent encoding of each cell type for each pair of latent variables. Eachpoint in each subplot represents a cell type, and cell types are colored by whether they areclassified as islet cells or not. The densities on the diagonal show the distribution of latent variablevalues for islet and non-islet cell types in each latent dimension. The left panel shows the latentvariables for BERRRI and the right plot shows the latent variables from a neural network.

**Additional file 3**. **Fig. S3** nn-PRRR coefficients for pancreatic cell types. Heatmaps showing the full coefficientmatrix UV^⊤^ for nn-PRRR (left is original, and right is on a log scale). Cell types are shown onthe rows and genes on the columns. In the left panel, white cells indicate values near zero,implying that this coefficient matrix is highly sparse.

**Additional file 4**. **Fig. S4** Marker genes identified by PRRR for pancreatic cell types. For each cell type, the tengenes with the highest coefficients in the matrix UV^⊤^ were extracted for each cell type. Somecell types share the same ten marker genes, which corresponds with our observation that the celltypes are largely overlapping in a PCA plot of the gene expression data (**Fig. S4**).

**Additional file 5**. **Fig. S5** PCA plot of pancreas scRNA-seq data. The first two principal components (PCs) areplotted. Each point corresponds to a single cell and is colored by its annotated cell type.

**Additional file 6**. **Fig. S6** nn-PRRR coefficients for GTEx eQTL mapping. Left: U matrix showing SNPs on therows and latent factors on the columns. Right: V matrix showing genes on the rows and latentfactors on the columns.

**Additional file 7**. **Fig. S7** Time complexity. Left: Time to fit each of the four models with varying sample sizes *n*.Right Left: Time to fit each of the four models with varying outcome dimensions *q*.

**Additional file 8**. **Fig. S8** The optimization problem for reduced-rank regression (RRR) and description of GTEx experiments.

## Data Availability

Pancreas scRNA-seq data were downloaded from GEO (GSE84133). https://www.ncbi.nlm.nih.gov/geo/query/acc.cgi?acc=GSE84133. GTEx bulk gene expression and SNP data were downloaded from the GTEx portal. https://gtexportal.org/home/datasets. Spatial gene expression data were downloaded from the 10x Genomics “Datasets” page. https://www.10xgenomics.com/resources/datasets.

## Abbreviations

RRR: reduced-rank regression
PRRR: Poisson reduced-rank regression
nn-PRRR: nonnegative Poisson reduced-rank regression
eQTLs: expression quantitative trait loci
MLE: Maximum likelihood estimate
MAP: Maximum *a posteriori*

## Code

Code is available at https://github.com/tianafitz/PRRR.
