# Supplementary figures and images for: A Poisson reduced-rank regression model for association mapping in sequencing data
Source: BMC Bioinformatics. 2022 Dec 8;23:529. doi: 10.1186/s12859-022-05054-6 (PMC9733401; doi:10.1186/s12859-022-05054-6)

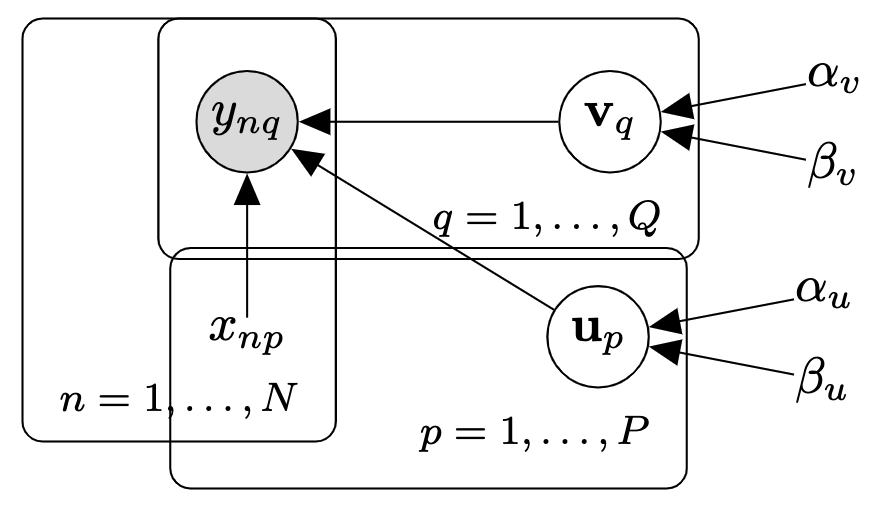

Supplement: Supplementary file 1 — Additional file 1. Fig. S1 Graphical model for PRRR and nn-PRRR. [file 12859_2022_5054_MOESM1_ESM.png]

a

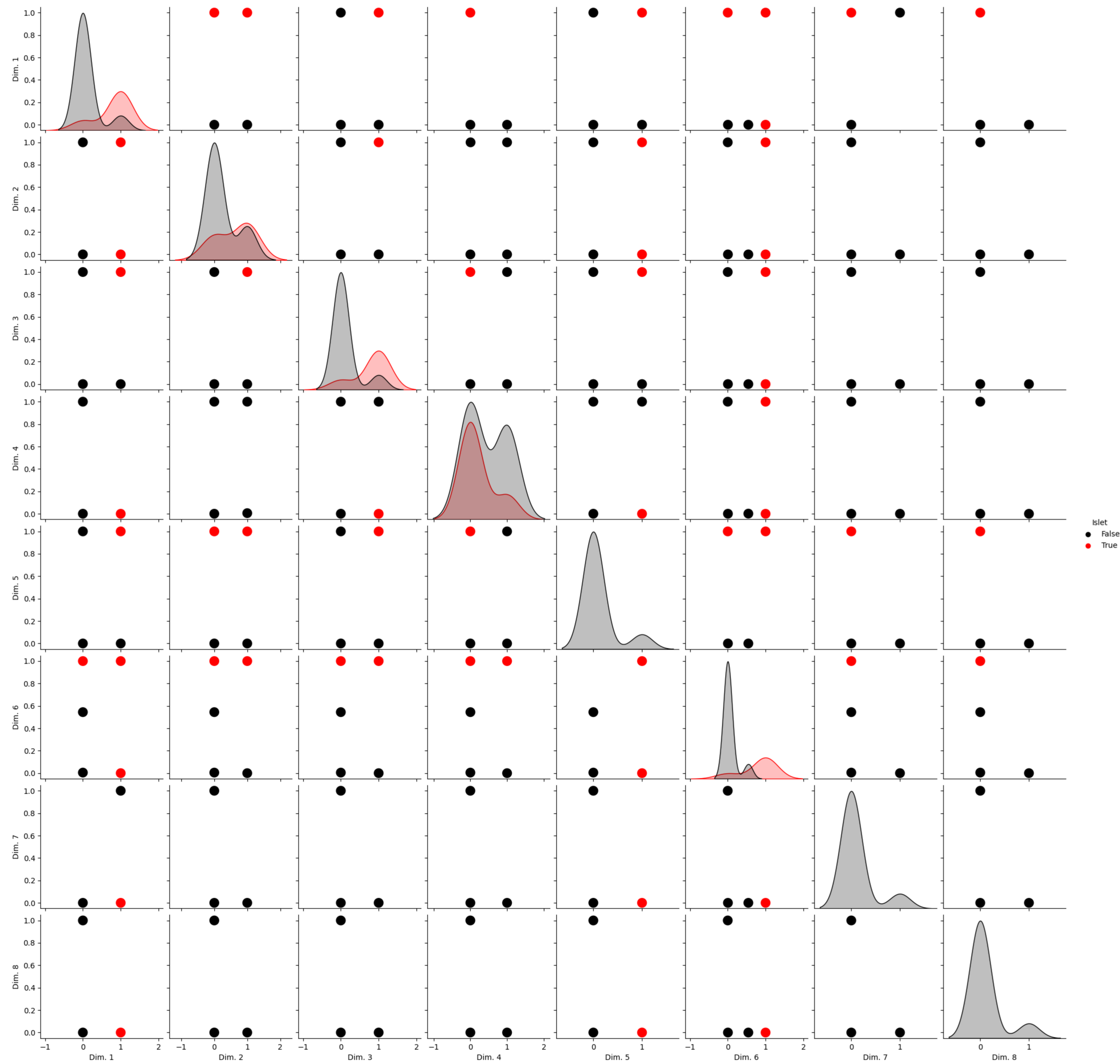

b

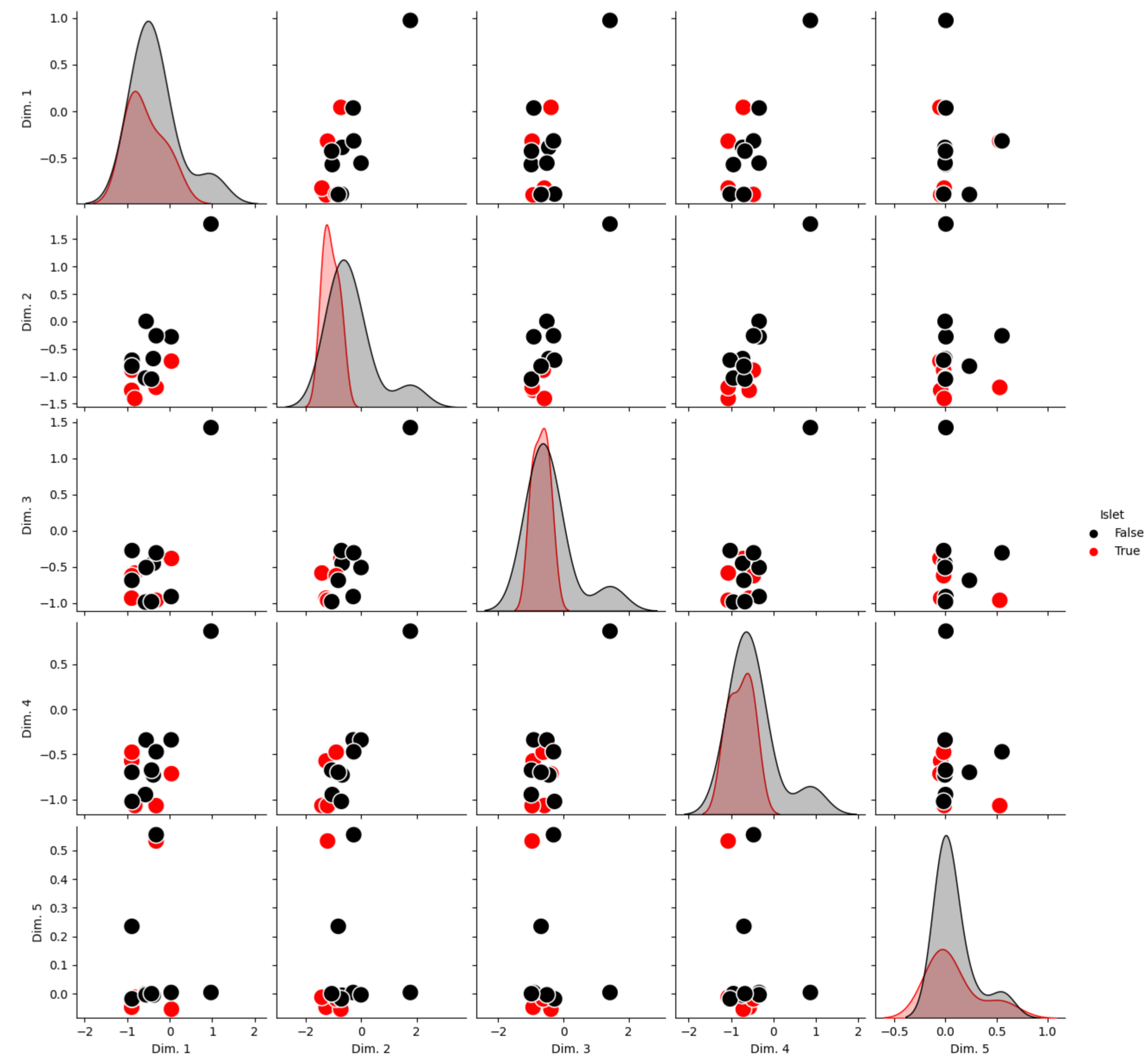

Supplement: Supplementary file 2 — Additional file 2. Fig. S2 BERRRI and a neural network approach fail to identify expression patterns in islet celltypes. Shown here is the latent encoding of each cell type for each pair of latent variables. Eachpoint in each subplot represents a cell type, and cell types are colored by whether they areclassified as islet cells or not. The densities on the diagonal show the distribution of latent variablevalues for islet and non-islet cell types in each latent dimension. The left panel shows the latentvariables for BERRRI and the right plot shows the latent variables from a neural network. [file 12859_2022_5054_MOESM2_ESM.pdf]

nn-PRRR coefficients

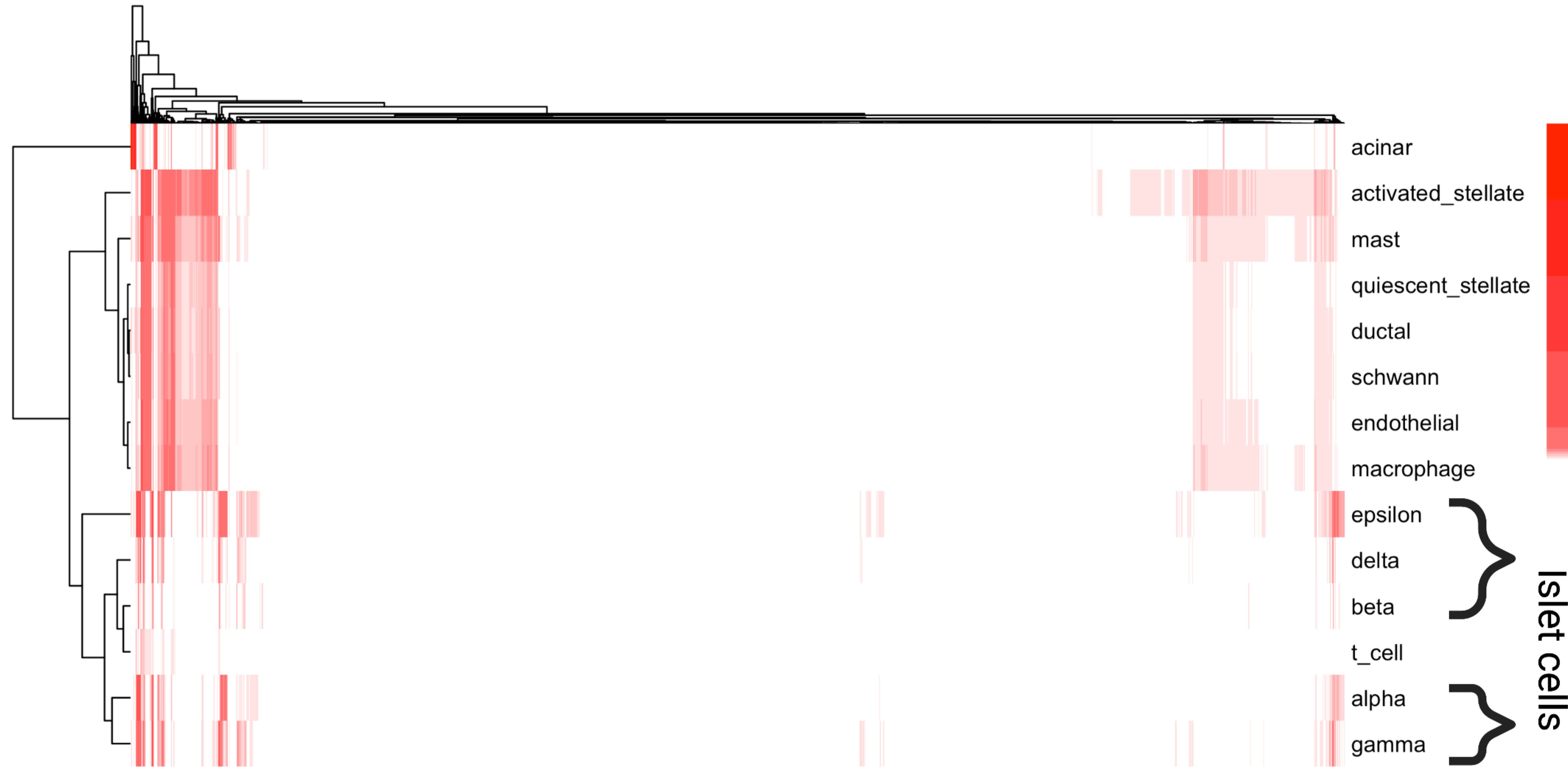

nn-PRRR coefficients

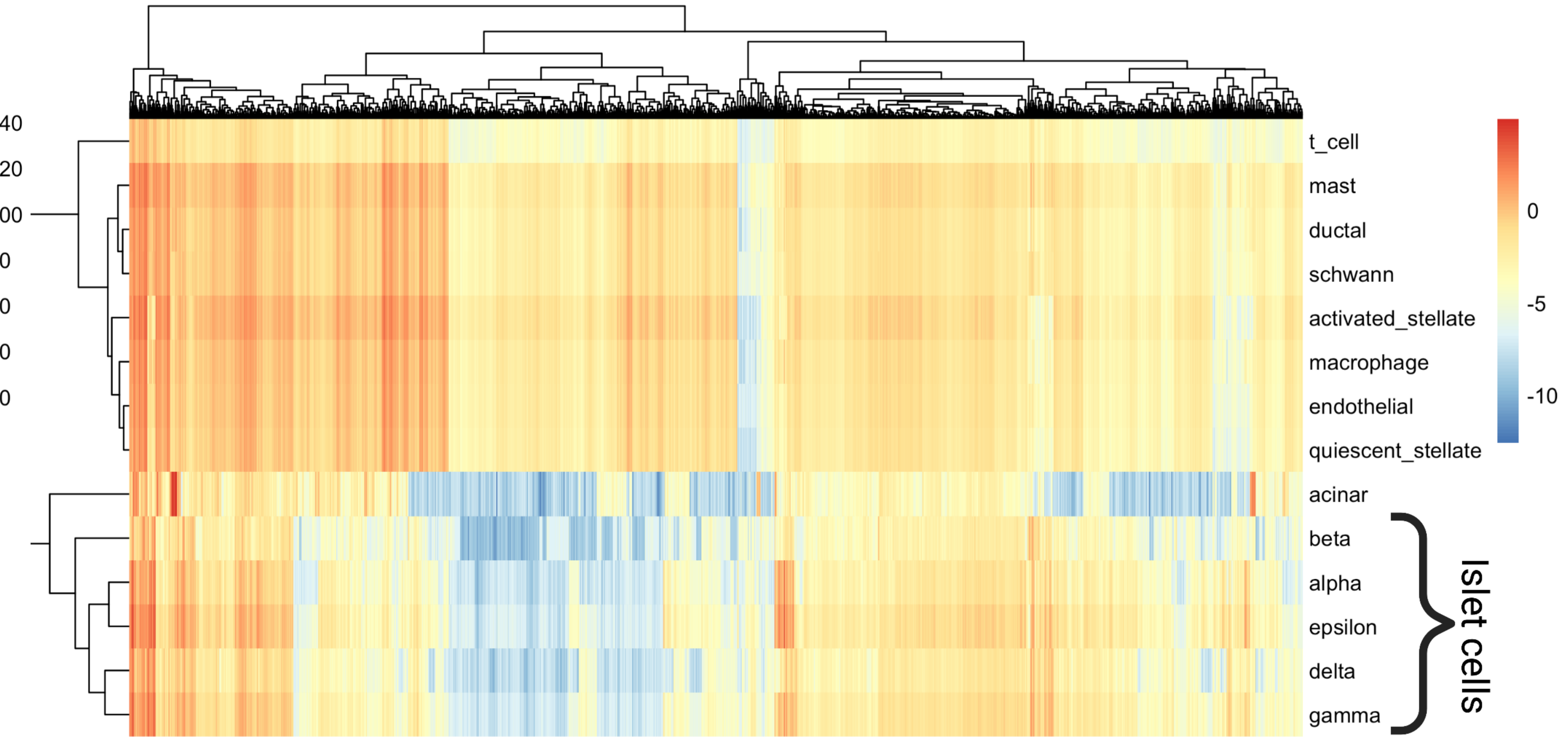

Supplement: Supplementary file 3 — Additional file 3. Fig. S3 nn-PRRR coefficients for pancreatic cell types. Heatmaps showing the full coefficientmatrix UV⊤ for nn-PRRR (left is original, and right is on a log scale). Cell types are shown onthe rows and genes on the columns. In the left panel, white cells indicate values near zero,implying that this coefficient matrix is highly sparse. [file 12859_2022_5054_MOESM3_ESM.pdf]

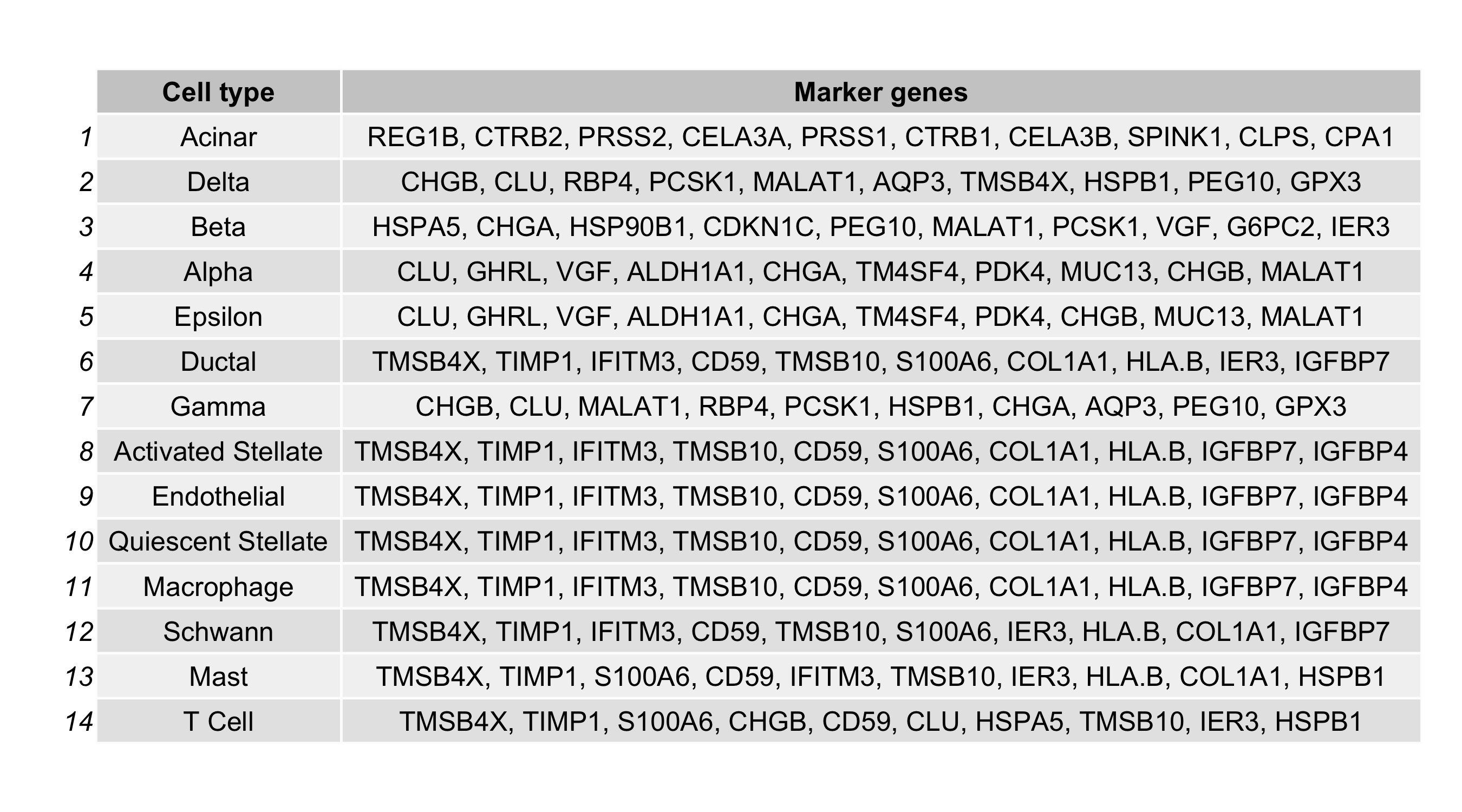

Supplement: Supplementary file 4 — Additional file 4. Fig. S4 Marker genes identified by PRRR for pancreatic cell types. For each cell type, the tengenes with the highest coefficients in the matrix UV⊤ were extracted for each cell type. Somecell types share the same ten marker genes, which corresponds with our observation that the celltypes are largely overlapping in a PCA plot of the gene expression data (Fig. S4). [file 12859_2022_5054_MOESM4_ESM.png]

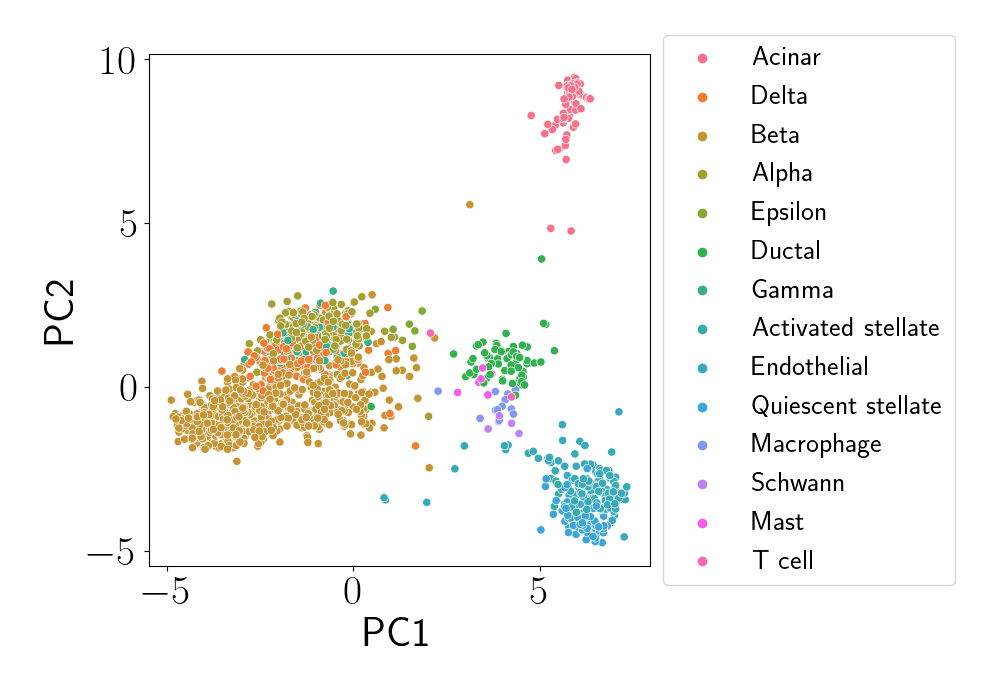

Supplement: Supplementary file 5 — Additional file 5. Fig. S5 PCA plot of pancreas scRNA-seq data. The first two principal components (PCs) areplotted. Each point corresponds to a single cell and is colored by its annotated cell type. [file 12859_2022_5054_MOESM5_ESM.png]

SNPs

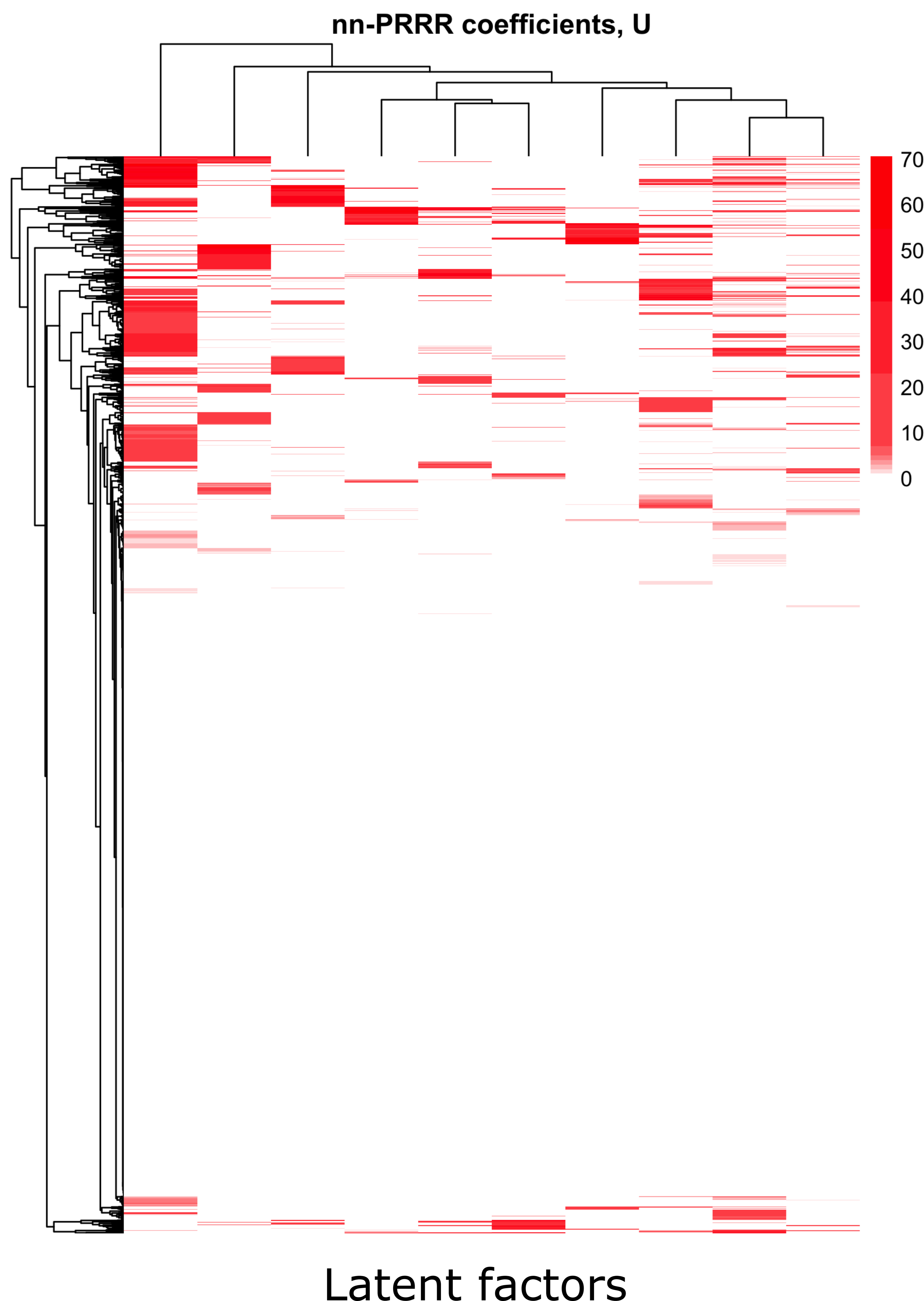

Genes

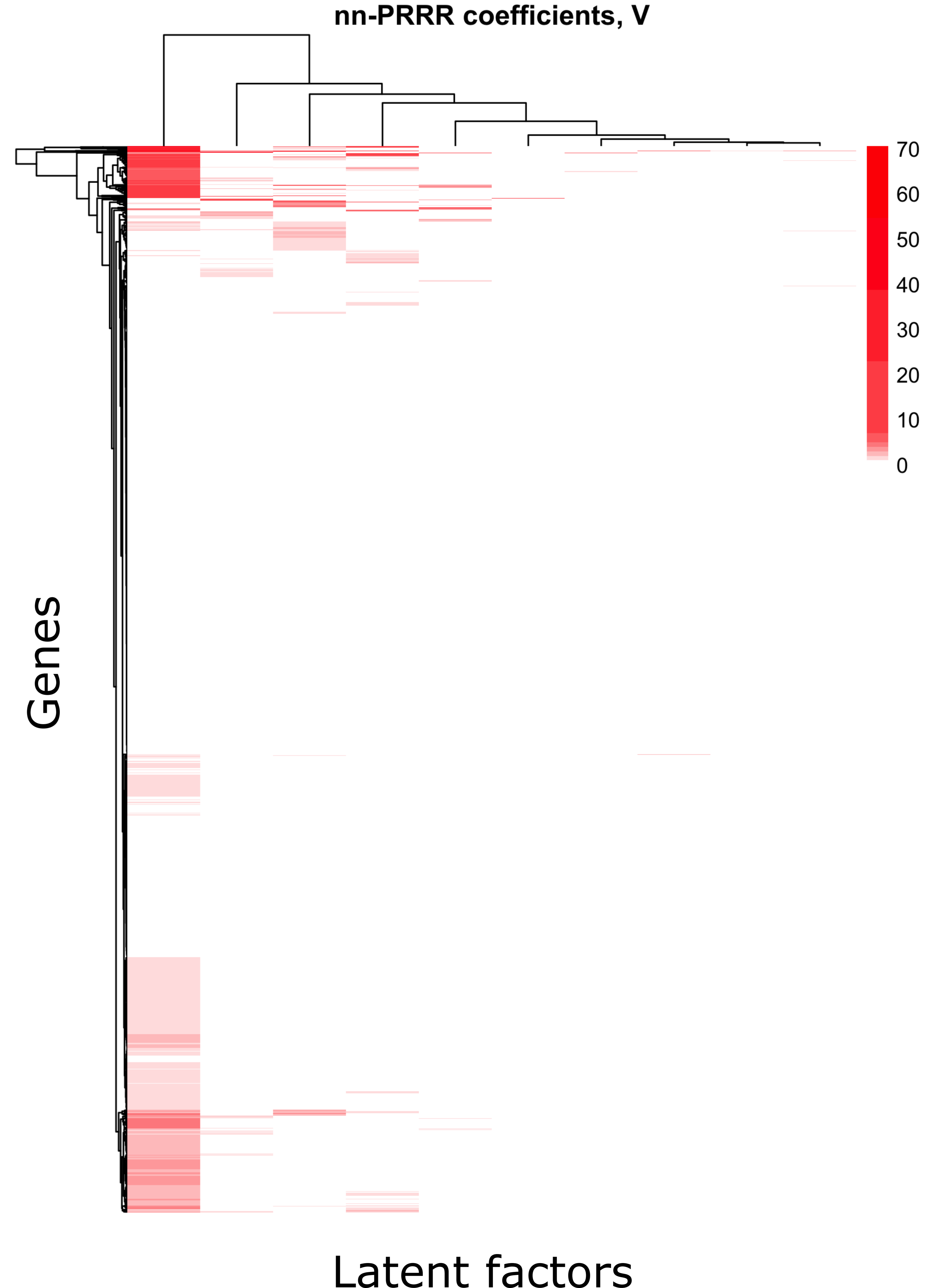

Supplement: Supplementary file 6 — Additional file 6. Fig. S6 nn-PRRR coefficients for GTEx eQTL mapping. Left: U matrix showing SNPs on therows and latent factors on the columns. Right: V matrix showing genes on the rows and latentfactors on the columns. [file 12859_2022_5054_MOESM6_ESM.pdf]

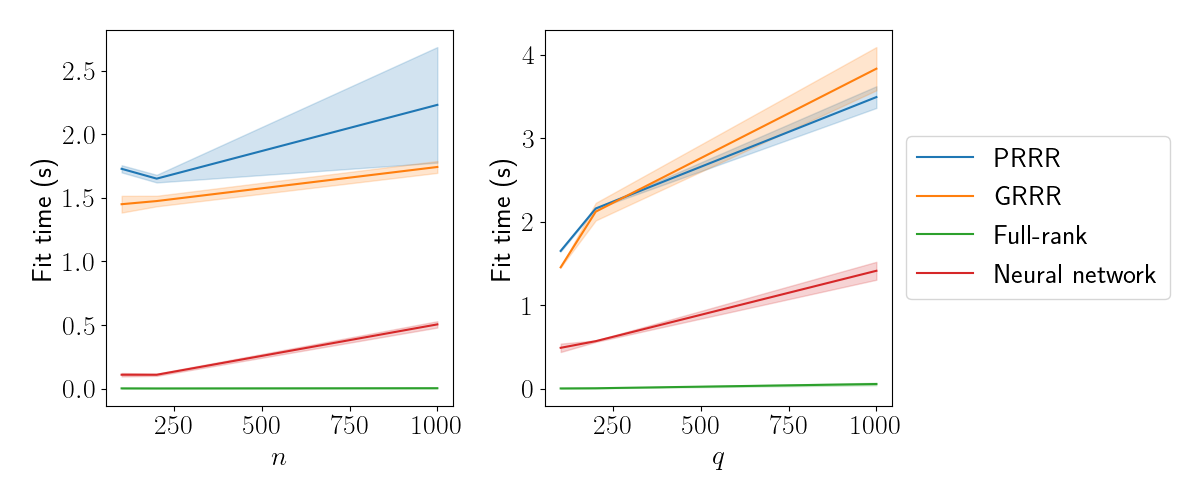

Supplement: Supplementary file 7 — Additional file 7. Fig. S7 Time complexity. Left: Time to fit each of the four models with varying sample sizes n.Right Left: Time to fit each of the four models with varying outcome dimensions q. [file 12859_2022_5054_MOESM7_ESM.png]
