# Supplementary material for: A Poisson reduced-rank regression model for association mapping in sequencing data
Source: BMC Bioinformatics. 2022 Dec 8;23:529. doi: 10.1186/s12859-022-05054-6 (PMC9733401; doi:10.1186/s12859-022-05054-6)
Supplement: Supplementary file 8 — Additional file 8. Fig. S8 The optimization problem for reduced-rank regression (RRR) and description of GTEx experiments. [file 12859_2022_5054_MOESM8_ESM.pdf]

Supplementary text

Appendix

0.1 RRR

The optimization problem for reduced-rank regression (RRR) is

$$\begin{aligned}
&\min_{\mathbf{B}} \|\mathbf{Y} - \mathbf{X}\mathbf{B}\|_2^2 \\
&\text{subject to } \operatorname{rank}(\mathbf{B}) \leq R,
\end{aligned}$$

where $\mathbf{X}$ is an $N \times P$ matrix of covariates, $\mathbf{Y}$ is an $N \times Q$ matrix of outcomes, and $\mathbf{B}$ is a $P \times Q$ coefficient matrix with rank at most R. When $R = \min(P, Q)$, we have the OLS solution,

$$\widehat{\mathbf{B}}_{OLS} = (\mathbf{X}^\top \mathbf{X})^{-1} \mathbf{X}^\top \mathbf{Y}.$$

We can write the RRR optimization program in terms of the OLS solution:

$$\begin{aligned}
&\min_{\mathbf{B}} \|\mathbf{Y} - \mathbf{X}\widehat{\mathbf{B}}_{OLS}\|_2^2 + \|\mathbf{X}\widehat{\mathbf{B}}_{OLS} - \mathbf{X}\mathbf{B}\|_2^2 \qquad (1) \\
&\text{subject to } \operatorname{rank}(\mathbf{B}) \leq R.
\end{aligned}$$

To see that these are equivalent, we can expand the loss function as follows.

$$\begin{aligned}
&\|\mathbf{Y} - \mathbf{X}\widehat{\mathbf{B}}_{OLS}\|_2^2 + \|X\widehat{\mathbf{B}}_{OLS} - \mathbf{X}\mathbf{B}\|_2^2 \\
=&\|\mathbf{Y} - \mathbf{X}(\mathbf{X}^\top \mathbf{X})^{-1}\mathbf{X}^\top \mathbf{Y}\|_2^2 + \|\mathbf{X}(\mathbf{X}^\top \mathbf{X})^{-1}\mathbf{X}^\top \mathbf{Y} - \mathbf{X}\mathbf{B}\|_2^2 \\
=&\mathbf{Y}^\top \mathbf{Y} - 2\mathbf{Y}^\top \mathbf{X}(\mathbf{X}^\top \mathbf{X})^{-1}\mathbf{X}^\top \mathbf{Y} + 2\mathbf{Y}^\top \mathbf{X}(\mathbf{X}^\top \mathbf{X})^{-1}\mathbf{X}^\top \mathbf{X}(\mathbf{X}^\top \mathbf{X})^{-1}\mathbf{X}^\top \mathbf{Y} \\
&\quad - \mathbf{Y}^\top \mathbf{X}(\mathbf{X}^\top \mathbf{X})^{-1}\mathbf{X}^\top \mathbf{X}\mathbf{B} - \mathbf{B}^\top \mathbf{X}^\top \mathbf{X}(\mathbf{X}^\top \mathbf{X})^{-1}\mathbf{X}^\top \mathbf{Y} + \mathbf{B}^\top \mathbf{X}^\top \mathbf{X}\mathbf{B} \\
=&\mathbf{Y}^\top \mathbf{Y} - \mathbf{Y}^\top \mathbf{X}\mathbf{B} - \mathbf{B}^\top \mathbf{X}^\top \mathbf{Y} + \mathbf{B}^\top \mathbf{X}^\top \mathbf{X}\mathbf{B} \\
=&(\mathbf{Y} - \mathbf{X}\mathbf{B})^\top (\mathbf{Y} - \mathbf{X}\mathbf{B}) \\
=&\|\mathbf{Y} - \mathbf{X}\mathbf{B}\|_2^2.
\end{aligned}$$

Now, the first term in the minimization problem in Equation (1) does not depend on B, so the problem becomes

$$\min_{\mathbf{B}} \|\mathbf{X}\widehat{\mathbf{B}}_{OLS} - \mathbf{X}\mathbf{B}\|_2^2.$$

Rewriting the objective, we have

$$\min_{\mathbf{B}} \|\mathbf{X}\widehat{\mathbf{B}}_{OLS} - \mathbf{X}\widehat{\mathbf{B}}_{OLS}\mathbf{B}\|_2^2.$$

Now, we can notice that this problem aligns with PCA. Let

$$\mathbf{\Sigma}_n = (\mathbf{X}\widehat{\mathbf{B}}_{OLS})^\top \mathbf{X}\widehat{\mathbf{B}}_{OLS} = \widehat{\mathbf{B}}_{OLS}^\top \mathbf{X}^\top \mathbf{X}\widehat{\mathbf{B}}_{OLS} = \mathbf{U}\Lambda\mathbf{U}^\top$$

be the eigenvalue decomposition of the covariance of the fitted values. Then we have

$$\widehat{\mathbf{B}}_{RRR} = \mathbf{U}_k,$$

where $\mathbf{U}_k = [\mathbf{u}_1, \cdots, \mathbf{u}_k]$ contains the eigenvectors corresponding to the top k eigenvalues.

0.2 GTEx metadata experiments

In an attempt to explore the organization of PRRR's low-dimensional space, we ran the model on a dataset containing gene expression counts along with patient metadata including height, weight, underlying conditions, and demographics. We wanted to study how features in the metadata are correlated with each other and with gene expression by analyzing the lower-dimensional matrices produced by the model. We were unable to recognize any apparent associations in the low-dimensional space, likely due to the small number of samples present in the data (about 200), and even less of these had all metadata points present.
